# Genetic study identifies novel genes in developmental dysplasia of the hip

**DOI:** 10.1038/s41413-026-00514-8

**Published:** 2026-03-31

**Authors:** Soichiro Yoshino, Shibo Chen, Ryosuke Yamaguchi, Taishi Kurakazu, Konstantinos Hatzikotoulas, Yoshinao Koike, Daisuke Inoue, Yusuke Kohno, Kan Sasaki, Hyonmin Choe, Shoji Baba, Toshihiko Hara, Juji Ito, Yaichiro Okuzu, Kyohei Shiomoto, Tomoyuki Nakamura, Gaku Koyano, Tomohiro Shimizu, Koichi Kinoshita, Eiji Takahashi, Takeshi Utsunomiya, Daisuke Hara, Taishi Sato, Shinya Kawahara, Ayumi Kaneuji, Takuaki Yamamoto, Daisuke Takahashi, Tetsuya Jinno, Tsutomu Kawano, Koji Goto, Michiaki Takagi, Taro Mawatari, Yutaka Inaba, Tetsuro Nakamura, Tamon Kabata, Satoshi Hamai, Goro Motomura, Lorraine Southam, J. Mark Wilkinson, Eleftheria Zeggini, Shiro Ikegawa, Yasuharu Nakashima, Chikashi Terao

**Affiliations:** 1 Laboratory for Statistical and Translational Genetics, RIKEN Center for Integrative Medical Sciences, Yokohama, Japan; 2 Department of Orthopaedic Surgery, Graduate School of Medical Sciences, Kyushu University, Fukuoka, Japan; 3 Institute of Translational Genomics, Helmholtz Zentrum München – German Research Center for Environmental Health, Neuherberg, Germany; 4 Munich Medical Research School (MMRS), Faculty of Medicine, Ludwig-Maximilians-Universität München, Munich, Germany; 5 Department of Orthopedic Surgery, Faculty of Medicine and Graduate School of Medicine, Hokkaido University, Sapporo, Japan; 6 Department of Orthopaedic Surgery, Graduate School of Medical Science, Kanazawa University, Kanazawa, Japan; 7 Department of Orthopaedic Surgery, Japan Community Healthcare Organization (JCHO) Kyushu Hospital, Kitakyushu, Japan; 8 Department of Orthopaedic Surgery, Yamagata Saisei Hospital, Yamagata, Japan; 9 Department of Orthopaedic Surgery, Yokohama City University, Yokohama, Japan; 10 Department of Orthopaedic Surgery, Hamanomachi Hospital, Fukuoka, Japan; 11 Department of Orthopaedic Surgery, Aso Iizuka Hospital, Iizuka, Japan; 12 Department of Orthopaedic Surgery, Yamagata University Faculty of Medicine, Yamagata, Japan; 13 Department of Orthopaedic Surgery, Kyoto University Graduate School of Medicine, Kyoto, Japan; 14 Department of Orthopaedic Surgery, Kyushu Rosai Hospital, Kitakyushu, Japan; 15 Department of Othopaedic and Spine Surgery, Fukuoka Children’s Hospital, Fukuoka, Japan; 16 Department of Orthopaedic Surgery, Dokkyo Medical University Saitama Medical Center, Koshigaya, Japan; 17 Department of Orthopaedic Surgery, Fukuoka University Faculty of Medicine, Fukuoka, Japan; 18 Department of Orthopaedic Surgery, Kanazawa Medical University, Kahoku, Japan; 19 Department of Orthopaedic Surgery, Kindai University Faculty of Medicine, Osakasayama, Japan; 20 School of Medicine and Population Health, The University of Sheffield, Sheffield, UK; 21 Technical University of Munich (TUM) and Klinikum Rechts der Isar, TUM School of Medicine, Munich, Germany; 22 Laboratory for Bone and Joint Diseases, RIKEN Center for Medical Sciences, Minato-ku, Tokyo, Japan; 23 Clinical Research Center, Shizuoka General Hospital, Shizuoka-shi, Shizuoka, Japan; 24 The Department of Applied Genetics, The School of Pharmaceutical Sciences, University of Shizuoka, Suruga-ku, Shizuoka, Japan

**Keywords:** Bone, Pathogenesis

## Abstract

Developmental dysplasia of the hip (DDH), a morphological abnormality of the hip joint, is a well-recognized risk factor for hip osteoarthritis (OA). Much remains unknown about the genetic factors of DDH and its subtypes. To further understand its genetic basis, we conducted genome-wide association studies (GWASs) using a total of 1 085 Japanese DDH cases (including 788 hip dysplasia cases without dislocation and 297 cases with dislocated hip) and 24 000 controls. Additionally, we meta-analyzed with United Kingdom (UK) DDH GWAS and the largest hip OA GWAS to date. We identified three genome-wide significant novel loci, *COL11A2*, *CALN1* and *TRPM7*, associated with hip dysplasia without dislocation. None of these signals were significant in dislocated hips, and additionally two of the signals had an opposite direction of association, suggesting distinct genetic architectures between the subtypes. The Japanese DDH GWAS identified five associated loci (*VEGF-C*, *FOXC1*, *SMC2*, *SLC38A4*, and *TRPM7*), and the trans-ancestry meta-analysis with UK revealed two loci (*COL11A1* and *GDF5*) supported by strong trans-ancestry genetic correlation (*r* = 1.0). In total, nine loci were identified for DDH and its subtypes, with hip dysplasia without dislocation showing distinct genetic signals from hip dislocation. The meta-analysis of DDH and hip OA identified five novel signals for hip OA. Susceptibility loci and heritability enrichment analyses implicated pathways involving bone formation, collagen type XI trimer, and chondrocyte development, as well as their gene regulation, in DDH. These findings enhance understanding of the genetic architecture and biological pathways underlying DDH, providing new insights into its relationship with OA.

## Introduction

Developmental dysplasia of the hip (DDH), formerly known as congenital dislocation of the hip, encompasses a spectrum of conditions ranging from infantile hip dislocation to hip dysplasia, characterized by acetabular dysplasia without dislocation, illustrating a continuum from a normal hip through dysplasia to dislocation.

DDH has long been recognized as a risk factor for secondary osteoarthritis (OA) due to cartilage wear.^1,2^ Additionally, individuals with DDH are more likely to develop hip OA and undergo total hip arthroplasty (THA) at a younger age, underscoring importance of clarification of the pathophysiology in DDH.^3^

DDH is a complex disease influenced by both genetic and environmental factors. Environmental factors associated with DDH are related to newborn limb positioning, diapering, holding, and swaddling (especially dislocated hip).^4,5^ Around 1972–1973 in Japan, Ishida and Yamamuro educated obstetricians, midwives, health nurses, and pregnant women about the natural leg position of newborns that would not interfere with acetabular development. They also advised manufacturers of diapers and baby clothes on appropriate clothing designs that allow newborns to move their lower limbs freely. This campaign aimed to prevent DDH and in fact has drastically reduced DDH incidence in Japan from 5.4% to 0.1%.^4,5^

Despite this dramatic decline brought about mainly by improvements of environmental factors, hip dysplasia, which accounted for about 80% of the causes of hip OA in a Japanese epidemiological survey in 2009, still accounts for more than 70% of the causes of OA in the Japanese population, supporting the importance of clarifying genetic factors of DDH and their impact on OA.^6,7^

The familial occurrence of DDH has been well recognized, suggesting the involvement of genetic factors in DDH.^8,9^ Stevenson DA et al. reported that the incidence of DDH in first-degree relatives to DDH cases was 12 times greater than that of people who did not have DDH relatives.^10^ Based on the data collected from approximately 300 cases of hip OA secondary to DDH, we found that a stronger genetic predisposition to DDH is associated with an earlier onset and faster progression of hip OA.^11^ Hatzikotoulas K et al. reported that autosomal single-nucleotide polymorphisms (SNPs) explained 55% of phenotypic variance (heritability) of DDH in UK, relatively strong heritability among complex traits.^12^ Regarding the susceptibility genes to DDH, candidate gene analyses have highlighted *GDF5* in hip OA secondary to DDH.^13^ Several genome-wide association studies (GWASs) on DDH have been reported, but the *GDF5* is the only locus so far with replicability across two or more independent cohorts, including the UK study.^12^ A moderate genetic correlation between DDH and primary hip OA without dysplasia was reported in the UK study. In fact, a shared susceptibility locus between primary hip OA and hip OA secondary to DDH was reported.^14^ Jacobsen et al. conducted their DDH GWAS using 408 Norwegian DDH cases and reported the *COL11A1* locus. While the *COL11A1* was not replicated in the meta-analysis with DDH cases alone, the meta-analysis with primary hip OA cohorts showed genome-wide significance in this region. Thus, while genetic components of DDH are largely unexplored, DDH genetic components seem to be shared with hip OA. In addition, no one has investigated the genetic differences between DDH subtypes.

In this study, we conducted the largest DDH GWAS meta-analysis to date, including 1 855 DDH cases (1 085 Japanese cases and 770 UK cases), to advance the understanding of the genetic factors of DDH, its subtypes and hip OA.

## Results

### DDH GWAS in Japanese

**Fig. 1:**
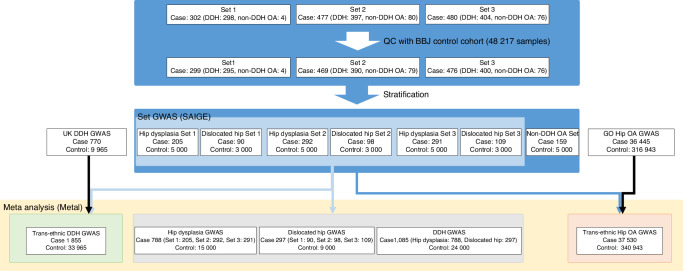
GWAS sets. Overview of GWAS sets. Set 1–3 was defined by the sample recruitment timing. 1 099 of the 1 259 cases were DDH and 160 were hip OA without DDH (non-DDH OA). A total of 788 hip dysplasia cases, 297 dislocated hip cases, 159 OA without DDH cases and 24 000 control samples remained after quality control

A total of 788 hip dysplasia cases, 297 dislocated hip cases, 159 OA without DDH cases, and 48 062 control samples remained after quality control. The variant quality control also retained 471 219 variants on the autosomes and 15 312 variants on the X chromosome (details, see Materials and Methods, Table 1 and Fig. 1). Following imputation, 40 401 910 variants passed quality control (Supplementary note).

**Table 1:** Study participants

Study		Hip dysplasia			Dislocated hip			Non-DDH OA GWAS set		
		Number	Female/Male	Age (s.d.) [year]	Number	Female/Male	Age (s.d.) [year]	Number	Female/Male	Age (s.d.) [year]
GWAS set 1	Case	205	184 / 21	61.8 (12.6)	90	81 / 9	61.5 (11.0)	159	123 / 36	72.6 (9.01)
	Control	5 000	2 299 / 2 701	66.0 (12.9)	3 000	1 362 / 1 638	66.4 (12.4)	5 000	2 189 / 2 811	66.2 (12.7)
GWAS set 2	Case	292	261 / 31	65.0 (12.7)	98	87 / 11	55.7 (23.6)	※As the total number of Non-DDH OA was small, we combined three groups together and performed one GWAS		
	Control	5 000	2 221 / 2 779	66.2 (12.7)	3 000	1 261 / 1 739	66.2 (12.5)			
GWAS set 3	Case	291	253 / 38	63.8 (14.9)	109	95 / 14	57.4 (19.6)			
	Control	5 000	2 182 / 2 818	66.2 (12.6)	3 000	1 325 / 1 675	65.9 (12.9)			

*DDH* developmental dysplasia of the hip, *GWAS* genome-wide association study, *s.d.* standard deviation, *OA* osteoarthritis

**Fig. 2:**
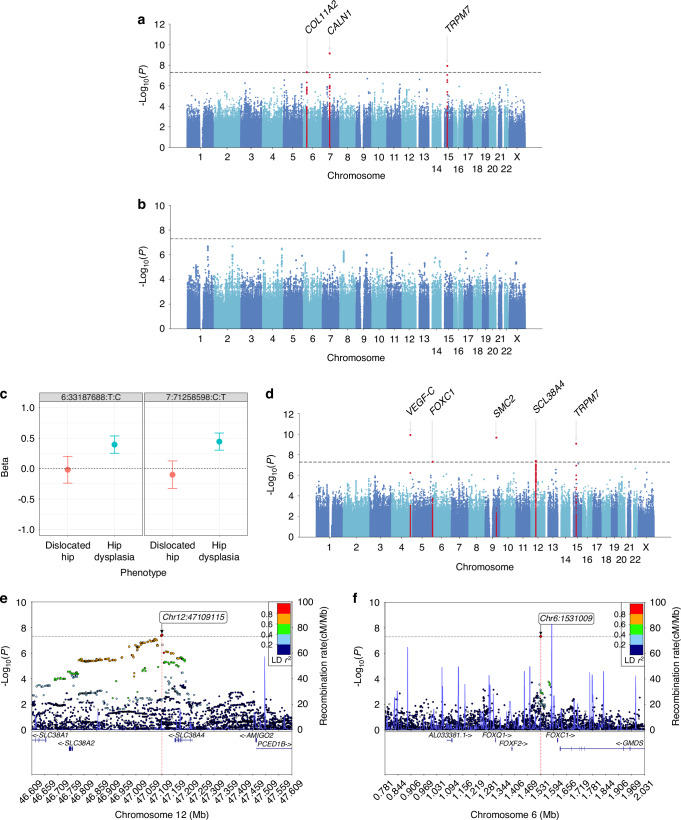
Manhattan plot of GWAS of DDH subsets, different genetic associations between the subsets and regional plots of meta-analysis of DDH in Japanese. **a** Manhattan plot of GWAS of Hip dysplasia. The y-axis shows the corresponding -log_10_ *P* values and the x-axis shows chromosome position along with variants. The horizontal grey dotted line indicates genome-wide significance threshold at *P* = 5.0 × 10^−8^. **b** Manhattan plot of GWAS of Dislocated hip. **c** Effect direction of two loci in dislocated hip and hip dysplasia. **d** Manhattan plot of meta-analysis of Hip dysplasia GWAS and Dislocated hip GWAS. **e** Regional plot of rs78572420. **f** Regional plot of rs146711505. The horizontal dotted line indicates genome-wide significance threshold at *P* = 5.0 × 10^−8^. Independent variants are colored purple. Other colored circles indicate pairwise linkage disequilibrium (LD). The strength of LD (*r*^2^) is presented in the upper right corner of each plot

In association studies, we first performed three GWAS each for hip dysplasia (set 1–3) and dislocated hip, depending on recruitment timing (Fig. 1), to assess subset-specific genetic signals in DDH. Subsequently, we performed a fixed-effect meta-analysis combining the three GWASs for hip dysplasia and dislocated hip, separately (Hip dysplasia: a total of 788 cases and 15 000 controls, Dislocated hip: a total of 297 cases and 9 000 controls; Fig. 1 and Fig. 2). As a result, the genomic inflation factors (λGC) were 1.04 and 1.02 for hip dysplasia and dislocated hip GWAS, respectively (Supplementary fig. 1a, b), suggesting almost no inflation of the statistics in the results. We identified three genome-wide significant loci for hip dysplasia GWAS (*P* < 5.0 × 10^−8^) (Fig. 2a and Table 2). Among the significant loci, rs1704995 in the MHC region on chromosome 6 (OR = 1.48 (1.29–1.71), *P* = 4.8 × 10^−8^) and located approximately 20 kb upstream of the *COL11A2* gene and was in moderate LD (*r*² = 0.62) with a missense variant rs9277934 in *COL11A2*. *COL11A2* is associated with type XI collagen disorders and skeletal and joint abnormalities.^15^ Due to the long and high LD structure in the MHC region, there was a possibility that HLA alleles or amino acids of HLA proteins could explain the signal. Thus, we conducted an HLA imputation and addressed potential associations of HLA alleles or amino acid residues. The analyses did not reveal any alleles or amino acid residues that showed a strong association with hip dysplasia (Fig. S2 and Table S1). Other significant signals were rs10241320, an intronic variant in *CALN1* (OR = 1.56 (1.35–1.79), *P* = 7.12 × 10^−10^), and rs7168702 (OR = 0.17 (0.09–0.31), *P* = 1.17 × 10^−8^), an intronic variant in *TRPM7*. *TRPM7* has been reported to be involved in bone regeneration^16^ and bone formation via the regulation of chondrogenesis.^17^ Conditional analysis did not reveal independent signals in these three loci. There were no genome-wide significant associations for dislocated hip considering its small sample size (Fig. 2b).

**Table 2:** Association results of DDH GWASs

rsID	CHR	POS	Gene	Allele1	Allele2	Freq1	OR(95%CI)	*P*-value	Direction	Het*P*-Val
Hip dysplasia										
rs1704995	6	33187688	*COL11A2*	T	C	0.817	1.48(1.29–1.71)	4.8 × 10^−8^	+++	0.21
rs10241320	7	71258598	*CALN1*	T	C	0.180	1.56(1.35–1.79)	7.1 × 10^−10^	+++	0.46
rs7168702	15	50959151	*TRPM7*	T	C	0.989	0.17(0.09–0.31)	1.2 × 10^−8^	−−−	0.69
DDH										
rs142273463	4	177942225	*VEGF-C*	A	G	0.994	0.12(0.06–0.23)	1.1 × 10^−10^	−−−−−−	0.19
rs146711505	6	1531009	*FOXC1*	T	TTG	0.234	0.73(0.66–0.82)	4.7 × 10^−8^	−−−−−−	0.62
rs147057560	9	107067481	*SMC2*	T	G	0.991	0.19(0.12–0.32)	2.1 × 10^−10^	−−−−−−	0.39
rs78572420	12	47109115	*SLC38A4*	A	G	0.775	0.74(0.66–0.82)	3.9 × 10^−8^	−−−−−−	0.070
rs7168702	15	50959151	*TRPM7*	T	C	0.990	0.20(0.12–0.33)	8.1 × 10^−10^	−−−+−−	0.30
Meta analysis JPN and UK DDH										
rs993471	1	103385373	*COL11A1*	A	G	0.639	1.23(1.14–1.33)	2.2 × 10^−8^	+++++++	0.59
rs143384	20	34025756	*GDF5*	A	G	0.686	1.39(1.29–1.50)	5.8 × 10^−17^	+++++++	0.33

*DDH* developmental dysplasia of the hip, *CHR* chromosome, *POS* position (position base pair in genome build hg19), *Allele1* effect allele, *Allele2* alternative allele, *Freq1* frequency for effect allele across this analysis, *OR* odds ratio, *CI* confidence interval, *P-value* meta-analysis *P*-value; Direction, summary of effect direction for each study (Hip dysplasia; GWAS set1/GWAS set2/GWAS set3, *JPN DDH* Japanese hip dysplasia GWAS set1/Japanese hip dysplasia GWAS set2/Japanese hip dysplasia GWAS set3/Japanese dislocated hip GWAS set1/Japanese dislocated hip GWAS set2/Japanese dislocated hip GWAS set3, Meta analysis JPN and UK DDH; UK-DDH GWAS /Japanese hip dysplasia GWAS set1/Japanese hip dysplasia GWAS set2/Japanese hip dysplasia GWAS set3/Japanese dislocated hip GWAS set1/Japanese dislocated hip GWAS set2/Japanese dislocated hip GWAS set3); HetPVal, *P*-value for heterogeneity statistic

We assessed genetic similarities and differences between hip dysplasia and dislocated hip and found contrasting results in some specific regions. rs1704995 in *COL11A2* and rs10241320 in *CALN1*, two of the three significant loci in hip dysplasia, showed opposite direction of effect in dislocated hip compared to hip dysplasia (Fig. 2c, Table S2). However, we found a very strong positive genetic correlation between hip dysplasia and dislocated hip, although it fell short of statistical significance due to limited power especially for dislocated hip (*r*^2^ = 1.0, *P* = 0.091) (table S3). Taken together, these suggest that while polygenic architecture is largely shared between the two subsets of DDH, there are genetic differences in a part of specific genetic loci.

Next, we conducted a meta-analysis to evaluate overall associations of DDH. As a result, λGC was 1.07 (Fig. S1C) and LDSC intercept of 1.05, suggesting little confounding bias in the results. We found five novel significant signals, including *TRPM7* found in hip dysplasia (Table 2 and Fig. 2d). Among the five signals, rs78572420 is located 50 kb downstream of *SLC38A4* (Fig. 2e) and is in high LD (*r*^2^ = 0.67) with a missense variant rs2429467 on *SLC38A4*. *SLC38A4* has been reported to be associated with a growth-regulated network of imprinted genes in the growth plate.^18^ rs146711505 was an intergenic variant located approximately 80 kb upstream of *FOXC1*, 140 kb downstream of *FOXF2*, and 210 kb downstream of *FOXQ1* (Fig. 2f). These genes have been implicated in bone formation, endochondral ossification, and the progression of OA through the regulation of pyroptosis.^19–21^ Conditional analyses did not find additional signals. Notably, none of the four significant signals excluding *TRPM7* were observed as significant in the subset GWASs (Table S4). Conversely, the two signals (excluding *TRPM7*) in hip dysplasia did not show genome-wide significance in DDH GWAS, supporting fine difference in genetic associations with overall genetic similarity between the subsets of DDH.

To confirm the robustness of our imputation results for rare variants, a secondary imputation analysis was performed using 62 samples with both WGS and array data. The analysis demonstrated high imputation quality (Rsq > 0.73) and confirmed that the three rare lead variants maintained genome-wide significance (Supplementary Note and Table S5).

To prioritize putative causal variants, we conducted a Bayesian statistical fine-mapping for significant loci using the FINEMAP software.^22^ Out of the five loci, four lead variants except *SLC38A4* showed high posterior probability (PP > 0.9) (Table 6).

Gene set enrichment analyses demonstrated nominally significant enrichment in the set related to collagen type XI trimer (*P* = 4.18 × 10^−5^) (Table S7). We conducted partitioning heritability enrichment analyses to investigate cell groups related to DDH. We observed nominally significant enrichment in the active enhancers of the connective/bone cell group (Fig. S3 and Table S8). We then analyzed each cell type belonging to this group and found significant enrichment of H3K27ac in chondrogenic differentiation cells (Table S9). Furthermore, in heritability enrichment analysis using the open chromatin region of three cell-types—chondrocytes, osteoblasts, and fibroblasts—we observed nominally significant and relatively strong enrichment in the open chromatin regions of chondrocytes (Fig. S4 and Table S10).

Except hip OA, there are almost no reports, either epidemiologically or genetically, on correlations between DDH and other diseases (regardless of bone and joint diseases or not). We assessed genetic correlations between DDH and complex traits, including various orthopedic diseases (Methods). We found nominally significant genetic correlations between DDH and keloids as well as hepatitis C (Table S11). Interestingly, we did not find strong genetic correlations of DDH with orthopedic diseases (Table S11).

### Meta-analysis with UK DDH GWAS

**Fig. 3:**
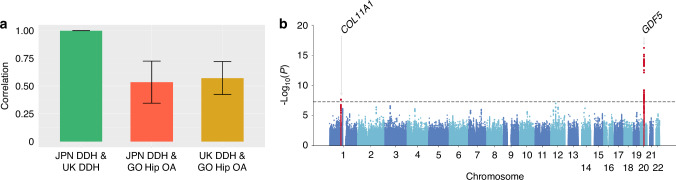
Genetic correlation of meta-analysis of DDH and hip OA and manhattan plot of trans-ancestry meta-analysis of DDH GWAS. **a** Genetic correlation between JPN DDH and UK DDH and between DDH and hip OA. The y-axis shows the genetic effect correlation. **b** The y-axis shows the corresponding –log_10_ *P*-values and the x-axis shows chromosome position along with variants. The horizontal grey dotted line indicates genome-wide significance threshold at *P* = 5.0 × 10^–8^

To further increase power of GWAS for DDH, we took advantage of UK data (Methods). We found a very strong genetic correlation between Japanese and UK populations (Methods, Fig. 3a and table S12). A meta-analysis revealed two genome-wide significant loci, namely, *COL11A1*, which is on chromosome 1 and different from *COL11A2* on chromosome 6 mentioned above, and *GDF5*, which has been previously reported by the UK GWAS (Fig. 3b, Fig. S1d, Table 2 and Table S13). The lead variants of these two loci overlapped with open chromatin regions in chondrocytes (Fig. S5), supporting the genetic associations. The lead variant rs993471 on *COL11A1* (MAF: Minor allele frequency (JPN/UK) = 0.35/0.38, OR = 1.23(1.14–1.33), *P* = 2.24 × 10^−8^) was in almost complete LD (*r*² = 0.998) with rs3753841, a missense variant predicted as possibly damaging (Methods). As is *COL11A2*, *COL11A1* is also associated with connective tissue or bone-related disorders, including some Type XI collagen disorders,^15^ lumbar disc herniation,^23^ and AIS.^24^ We also found that rs4911494, a missense variant in *GDF5*, was in very strong LD (*r*² = 0.92) with the lead variant, rs143384 (MAF: (JPN/UK) = 0.23/0.41, OR = 1.39(1.29-1.50), *P* = 5.78×10^−17^).

Gene Set Enrichment Analysis revealed the set related to collagen type XI trimer (*P* = 8.3 × 10^−7^), embryonic skeletal system morphogenesis (*P* = 2.7 × 10^−5^), regulation of bone development (*P* = 5.9 × 10^−4^) and embryonic skeletal system development (*P* = 1.9 × 10^−4^) as nominally significant (Table S14).

### Meta-analysis with GO hip OA GWAS summary statistics

**Fig. 4:**
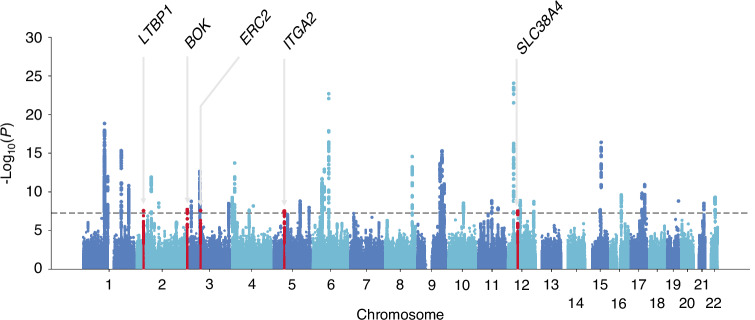
Manhattan plot of meta-analysis of DDH and hip OA. The y-axis shows the corresponding –log_10_ *P*-values and the x-axis shows chromosome position along with variants. The horizontal grey dotted line indicates genome-wide significance threshold at *P* = 5.0 × 10^−8^

Since DDH is a strong risk factor of hip OA, to maximize statistical power to detect novel signals in hip OA, we used summary statistics of European hip OA in GO,^25^ international consortium of osteoarthritis, and meta-analyzed with Japanese DDH. Aside from *SLC38A4*, showing the significant signal in Japanese DDH, we found additional four signals, namely, *LTBP1*, *BOK*, *ERC2* and *ITGA2*, all of which did not reach statistical significance in GO hip-OA GWAS alone (Fig. 4, Fig. S1e, Table 3 and Table S15).

**Table 3:** Association results of meta analysis between JPN DDH or non-DDH hip OA and GO hip OA

RSID	CHR	POS	Gene	Allele1	Allele2	Freq1	OR(95%CI)	*P*-value	Direction	Het*P*-Val
rs2167973	2	33555177	*LTBP1*	A	C	0.87	0.93(0.90–0.95)	2.7 × 10^−8^	−−−−+−++	0.53
rs876531	2	242500662	*BOK*	T	C	0.45	0.95(0.93–0.97)	1.9 × 10^−8^	−−−−−++−	0.28
rs1519038	3	56066102	*ERC2*	T	C	0.79	0.94(0.92–0.96)	2.6 × 10^−8^	−+−−−−+−	0.19
rs35233	5	52290880	*ITGA2*	A	G	0.12	0.93(0.90–0.95)	2.8 × 10^−8^	−−−−+−−+	0.55
rs2408618	12	47106260	*SLC38A4*	A	T	0.22	1.34(1.21–1.49)	3.0 × 10^−8^	++++++++	0.15

*DDH* developmental dysplasia of the hip, *OA* osteoarthritis, *GO* Genetics of Osteoarthritis, *CHR* chromosome, *POS* position (position base pair in genome build hg19), *Allele1* effect allele, *Allele2* alternative allele, *Freq1* frequency for effect allele across this analysis, *OR* odds ratio, *CI* confidence interval, *P*-value meta-analysis *P*-value, Direction, summary of effect direction for each study (GO hip OA GWAS/Japanese hip dysplasia GWAS set1/Japanese hip dysplasia GWAS set2/Japanese hip dysplasia GWAS set3/Japanese dislocated hip GWAS set1/Japanese dislocated hip GWAS set2/Japanese dislocated hip GWAS set3/Japanese non-DDH hip OA GWAS), *HetPVal*, *P*-value for heterogeneity statistic

To assess the extent to which hip OA and DDH share genetic components, we conducted a trans-ancestry genetic correlation analysis and found a moderate correlation between the two (Fig. 3a and Supplementary table 16). When we limited our analysis to the 45 lead variants identified in the GO hip OA GWAS and compared with the beta values in the Japanese DDH, we found that the directions of the effect were consistent for 30 out of 45 variants (Binomial *P* = 3.6 × 10^−2^) and observed a moderate correlation of effect sizes (Spearman correlation = 0.53, *P* = 2.4 × 10^−4^, Fig. S6). These support that genetic analyses of DDH can contribute to understanding the basis of hip OA.

## Discussion

DDH still accounts for more than 70% of the causes of hip OA in Japan, even though the incidence of dislocated hip has been reduced by the prevention campaign.^7^ Considering high prevalence of DDH in subjects with family history, genetic factors play fundamental roles in the pathogenesis of DDH. Since many components, including genetic and non-genetic, are involved with dislocation, we assumed that hip dysplasia with and without dislocation might have a different genetic background, at least in part. Therefore, we conducted separate GWASs for hip dysplasia with and without dislocation at first, and then performed a meta-analysis as a DDH GWAS.

*COL11A2*, a locus significantly associated with hip dysplasia, encodes one of the two alpha chains of type XI collagen. Variants in *COL11A2* can cause skeletal abnormalities such as otospondylomegaepiphyseal dysplasia (OSMED), fibrochondrogenesis, and Stickler syndrome, which often lead to early onset of OA.^15^ Interestingly, rs9277935, in moderate LD (*r*^2^ = 0.34) with the lead variant in the current study, was shown to regulate the expression and chondrogenic properties of *COL11A2* in a Chinese DDH study.^26^ Additionally, *COL11A2* is known for its association with various musculoskeletal conditions. *COL11A2* has been identified as a methylation quantitative trait locus (mQTL) in cartilage from hip and knee OA,^27^ a differentially expressed gene in damaged knee osteoarthritic cartilage,^28^, and a candidate gene for vertebral malformations and congenital scoliosis.^29^ These findings underscore involvement of *COL11A2* in various diseases characterized by degeneration and morphological abnormalities of bone and cartilage tissues.

We also identified *COL11A1* as a susceptibility locus, which encodes the other alpha chain of type XI collagen, through a trans-ancestry meta-analysis of DDH. *COL11A1*, as with *COL11A2*, plays a crucial role in bone development and cartilage formation and is associated with some skeletal disorders.^15^ In addition to the numerous studies linking *COL11A1* to hip osteoarthritis (hip OA),^25,30–32^ *COL11A1* was reported for its potential link to DDH. A Norwegian DDH GWAS found this region associated with DDH and hip OA secondary to DDH only in a discovery data set.^14^ We found a missense variant, rs3753841, in strong LD (*r*^2^ = 0.998) with the lead variant, suggesting that functional alterations of COL11A may potentially contribute to the pathogenesis of DDH. The involvement of *COL11A1* and *COL11A2*, which are associated with the early onset of OA, in the susceptibility to DDH or its subtype suggests that these genes influence the onset and progression of DDH and OA. The replication of the association of *GDF5* with DDH in our study supports the validity of our analyses (Table 2 and Table S13).

In addition to these two type-XI collagen-related genes, we discovered other novel signals in DDH. Both *FOXF2* and *FOXC1* are implicated in bone formation via Wnt2b/β-catenin signaling and endochondral ossification,^19,20^, and *FOXQ1*, a gene near *FOXF2/FOXC1* is suggested to be involved in the progression of OA through the regulation of pyroptosis.^21^ Although there are few reports on functions of *SLC38A4*, the expression of *SLC38A4* increases during the growth process of long bones,^18^ supporting the genetic associations in the current study.

We should be cautious about associations of rare variants in three among the nine signals with DDH or its subtype, namely, *TRPM7*, *VEGF-C*, and *SMC2*. Further recruiting case subjects and extensive validation would consolidate these associations. As one such validation, we conducted WGS-based secondary imputation analysis, which confirmed high Rsq values for all three variants consistent with the primary imputation. All three variants retained genome-wide significance in the secondary imputation, while we should further validate the association of *VEGF-C* locus variant (rs142273463), considering its very low number of heterozygote carriers in our WGS. While these associations require further validation, there is functional evidence in support of their involvement in joint degeneration and morphological abnormalities. *VEGF-C* is suggested to be the most critical regulating factor of the synovial lymphatic system (located in the subintimal layer of the synovium), promoting lymphatic drainage, restoring intra-articular homeostasis, and suppressing chronic inflammation within the joint and the progression of OA.^33,34^ *TRPM7* is reported to mediate bone and cartilage formation or development by regulating fluctuations of Mg and Ca ions.^16,35^

The meta-analysis of GWAS for general hip OA, including both primary and secondary to DDH, assuming common pathological mechanisms, identified five new loci. *ITGA2* encodes the alpha subunit of a transmembrane receptor for collagens and related proteins. It is related to apoptotic pathways in synovial fibroblasts and integrin pathway. Itga2 knockout mouse model of rheumatoid arthritis showed loss of α2β1 integrin and reduced levels of secreted matrix metalloproteinase 3 (MMP-3), which suppressed joint inflammation and cartilage destruction.^36^ *LTBP1*, together with *LTBP3* and TGF-b signaling *FBN2*, is thought to influence the pathogenesis of OA through its involvement in the progression of fibrosis.^25,37^

In addition to functions of GWAS susceptibility genes related to biology of bone and chondrocyte, polygenic signals in the DDH GWASs showed enrichment in the active enhancers of the connective/bone cell group and the open chromatin region of chondrocytes. Thus, both GWAS significant and non-significant signals seem to converge to the regulation of gene expressions or functions in bone and cartilage tissues. These results would make the current study convincing, while further expansion of GWAS is favorable.

To further interpret our GWAS signals, we integrated them with publicly available functional genomic datasets (Supplementary Note, Table S17 and Table S18). Analysis of chondrocytes-derived data from talar cartilage^38^ revealed that rs6088815 and rs2425066, both in high LD with the *GDF5* lead variant from our UK meta-analysis, overlapped with eQTLs, suggesting that DDH risk may be mediated through *GDF5* regulation in chondrocytes. No significant overlap was found with condition-specific chromatin accessibility or 3D chromatin structure, likely reflecting cell type– or tissue-specific differences in gene regulation. In addition, several lncRNAs identified from acetabular bone and cartilage^39^ were located near our GWAS signals (Table S18), highlighting potential novel regulatory regions.

These findings underscore the need for future DDH-specific multi-omics studies, integrating genetic data with tissue-specific gene expression, chromatin accessibility, and spatial chromatin structure, especially in chondrocytes, to fully elucidate the functional mechanisms underlying this complex disorder.

There are some limitations in our study. First, although the GWASs in the current study are the largest so far, statistical power is still insufficient. Additional samples are necessary to further clarify the pathogenesis of DDH, especially to conclude genetic differences between the subsets of DDH. Second, although we lacked gene expression data in cartilage, bone, or soft tissue of the hip joint from our own cohort, we partially addressed this limitation by integrating publicly available datasets, which provided relevant expression, chromatin accessibility, and lncRNA information. While these resources helped to support our interpretation of GWAS signals, the absence of single-cell RNA sequencing or bulk RNA-seq data from our own DDH-related tissues remains a limitation, as such data would allow more precise detection of subtype-specific gene expression patterns. Third, since there are differences in the definition of DDH between the UK and Japan, these differences might affect trans-ancestry meta-analysis results via misclassification bias. While this would be addressed in future studies, based on the very high genetic correlation, we assume that the bias is minimal. Fourth, the lack of replication or attenuation of some Japanese-specific loci in the trans-ancestry meta-analysis is primarily attributable to population-specific genetic architectures, specifically differences in MAF and LD patterns. For most of the attenuated variants, the primary reason was lack or rarity of the lead variants in UK (MAF_UK = 0.012 vs MAF_JPN = 0.222 for rs78572420 on chromosome 12), which resulted in no or unstable association estimates. However, the non-replication of the chromosome 6 lead variant (rs146711505) was an exception where the MAF was comparable between EAS and EUR populations (Table S19). This attenuation was due to a marked difference in LD structure; high-LD European proxies (*r*^2^ > 0.8) showed low LD in the Japanese (*r*^2^: 0.28 − 0.29; see Supplementary Note and Table S20). These observations suggest that differences in genetic architecture (MAF and LD), rather than a lack of true association, largely contribute to the non-replication of signals across populations.

In conclusion, this study identified susceptibility loci to DDH and hip OA and candidates of responsible genes in the loci. Our study showed distinct genetic loci between subsets of DDH. This study will provide a genetic basis to facilitate future studies in order to elucidate the pathogenesis of DDH and hip OA and to develop new therapeutic strategies.

## Materials and Methods

### Ethical approval

Informed consent was obtained from all participants. This multi-institutional study was approved by the Central Institutional Review Board of Kyushu University or the Institutional Review Board in each institution and RIKEN.

The United Kingdom Household Longitudinal Study has been approved by the University of Essex Ethics Committee and informed consent was obtained from every participant.

### Subjects of this study

#### Japanese phenotype definition

A total of 1 259 subjects with DDH or hip OA were collected throughout Japan. Participants retrospectively underwent clinical assessment questionnaires by orthopedic surgeons. Diagnosis of DDH was made when any one of the following three conditions (for cases ≥ 10 years old) or two conditions (for cases < 10 years old) measured on anteroposterior hip radiographs of either the left or right hip was met.^40^ Cases ≥ 10 years old: Center Edge (CE) angle ≤ 20°, Sharp angle ≥ 45°, and Acetabular roof obliquity ≥ 15°. Cases < 10 years old: CE or Ombredanne-Epiphyseal (OE) angle ≤ 15° and Acetabular index ≥ 30°. Cases < 5 years old: OE angle ≤ 5° and Acetabular index ≥ 30°. The radiographic diagnosis of hip dislocation was defined as decentered/eccentric femoral head in cases < 10 years old, otherwise defined according to the Crowe classification: Crowe Group ≥2.^41^ A dislocated hip case was defined by this radiographic diagnosis or by a history of treatment for infantile hip dislocation revealed by the clinical assessment questionnaire. Hip OA was diagnosed when any one of the hip joints was less than 2 mm on anteroposterior hip radiographs. According to these diagnostic criteria, 1 099 of the 1 259 cases were DDH and 160 were hip OA without DDH (non-DDH OA). Out of the 1 099 DDH cases, 300 were dislocated hip cases and 799 were hip dysplasia (without dislocation) cases (Fig. 1 and Table 1). Controls were individuals from BioBank Japan (BBJ)^42,43^ that were not overlapping among the seven data sets (as described below).

### UK phenotype definition

The UK collection was approved by the National Research Ethics Service in England (NRES 12/YH/0390, 30 October 2012). The DDH samples were identified from the National Joint Registry cohort, which at the time included 5 411 adult individuals living in England who had undergone a hip replacement surgery for DDH. They were surgeon-confirmed DDH, diagnosed using the Crowe classification, which grades the severity of hip dislocation. All 5 411 patients were approached to provide DNA for study, from whom approximately 850 agreed to participate. A total of 770 DDH patients were included after implementing sample quality control. The control sample comprised 9 965 participants from the UK Household Longitudinal Study. All participants were European ancestry.

### Genotyping and imputation

Genotyping and imputation methods are summarized in the online supplemental text.

### Association analysis

The details of association analysis are described in online supplementary text. In brief, we performed a total of seven Japanese GWASs using six different DDH case groups separated by three genotyping batches and the presence or absence of dislocated hip, and one OA case group without DDH. This means that our GWAS data consists of seven sets: Hip dysplasia GWAS-1, Hip dysplasia GWAS-2, Hip dysplasia GWAS-3, Dislocated hip GWAS-1, Dislocated hip GWAS-2, Dislocated hip GWAS-3, and non-DDH OA GWAS. There was no overlap of individuals among the seven Japanese GWAS sets. Japanese GWASs and the UK DDH GWAS were performed using a generalized linear mixed model with SAIGE version 0.35.8.3 and 0.44.6.4, respectively.^44^

### Meta-analysis

We performed 3 meta-analyses for DDH, hip dysplasia and dislocated hip with an inverse variance method under a fixed effect model using METAL.^45^ Genomic control was not performed on all datasets. Only variants (with a minimum MAF ≥ 0.005) common to the datasets used in the meta-analyses were considered.

### HLA imputation and association analysis

To evaluate whether the association near *COL11A2* in the MHC region was attributable to specific HLA alleles or amino acid variants, we performed HLA imputation using the HLA-TAPAS pipeline (https://github.com/immunogenomics/HLA-TAPAS) as previously described by Luo et al. ^46^ Genotype dosages from post-imputation GWAS data were used as input, and HLA alleles were imputed at four-digit resolution for classical class I (HLA-A, -B, -C) and class II (HLA-DQA1, -DQB1, -DRB1) loci, using a multi-ancestry reference panel derived from high-coverage whole-genome sequencing.

Association analyses were performed for imputed HLA alleles and inferred amino acid polymorphisms using logistic regression, adjusting for sex and the first ten principal components. Analyses were restricted to alleles and residues with a minor allele frequency (MAF) > 1%.

### Conditional association analysis

We used the distance-based approach to determine independently significant loci. We defined the variant with the lowest *P*-value within each locus as the lead variant. We defined an associated locus as 1 Mb window around the lead variant. We conducted conditional analyses of GWAS set 1–3 separately and integrated the results using METAL (The detailed method follows the Meta-analysis section described earlier.). We repeated this process until the top associated variants fell below the genome-wide significance threshold (*P* < 5.0 × 10^−8^).

### Estimation of phenotypic variance

The heritability of DDH was estimated using LDSC software.^47^ The variance explained by the variants was calculated based on a liability threshold model assuming the prevalence of DDH to be 13.4%.^48^

### Bayesian statistical fine-mapping analysis

To prioritize causal variants in disease susceptibility loci, we conducted a fine-mapping analysis using FINEMAP v1.3 software^22^ and LD matrices calculated by 1KGP3 EAS and JEWEL 3 K data. We assumed one causal signal in the ±1 Mb region from both ends of significant variants at each significant locus. We calculated a posterior probability (PP) in which each genetic variant was the true causal variant. Then, we ranked the candidate causal variants in descending order of their PPs and created a 95% credible set of causal variants by adding the PPs of the ordered variants until their cumulative PP reached 0.95.

### Gene set enrichment analysis

We conducted a gene set enrichment analysis using FUMA.^49^ Because variants often act on the closest genes,^50^ we selected genes on a distance basis using the following criteria: genes (i) located within 1 Mb and (ii) the five closest to the lead variants in each significant locus.

### Partitioning heritability enrichment analysis using 220 cell-type-specific annotations

We conducted stratified LD score regression using 220 cell-type-specific annotations of four histone marks (H3K4me1, H3K4me3, H3K9ac, and H3K27ac).^51^ We assessed heritability enrichment in histone marks of these ten cell type groups of the 220 annotations, as previously described.^52^ We excluded variants within the major histocompatibility complex (MHC) region (chromosome 6: 25–34 Mb). We defined significance of heritability enrichment based on FDR < 0.05.

### Genetic correlation analysis

We estimated the genetic correlations using a bivariate LD score regression.^47^ The GWASs included in this analysis were GWAS for 42 diseases,^53^ 60 quantitative traits,^54^ adolescent idiopathic scoliosis,^55^ ossification of the posterior longitudinal ligament,^56^ and knee osteoarthritis (in-house data). We excluded variants in the MHC region. We set the significance threshold for genetic correlations as FDR < 0.05.

### Trans-ancestry genetic correlation analysis

To estimate the genetic correlation of GWAS results in different populations, the popcorn software was used.^57^ East Asian and European data of the 1KG were used to compute cross-population scores.^58^

### Comparison of the effect sizes between DDH and hip OA

We evaluated the correlations of the effect sizes of variants between DDH and hip OA. We used the results of the GWAS meta-analysis of DDH and the European GWAS of hip OA.^25^ First, we extracted 6 263 042 variants with MAF ≥ 0.01, shared between the meta-analyses for DDH and hip OA. Next, we conducted LD pruning of the variants for the variant pairs in LD (*r*^2^ ≥ 0.3) using 1KGP3 East Asian / European and JEWEL 3 K data by PLINK. Finally, we used 552 938 variants of DDH summary statistics and 462 842 variants of hip OA summary statistics in subsequent analyses. We calculated the correlation using R software (version 4.0.2).

### Reporting summary

Further information on research design is available in the Nature Research Reporting Summary linked to this article.

## Supplementary information

Supplementary material

Reporting summary

Editorial policy checklist

## Data Availability

The GWAS and Meta-analysis summary statistics generated in this study will be available after acceptance via the website of the Japanese ENcyclopedia of GEnetic associations by Riken (JENGER, http://jenger.riken.jp/en/). Source data are provided with this paper. The remaining data are available within the article, Supplementary Information.

## Code availability

For the codes for statistical analyses, we followed the publicly available codes and the instructions, which are provided by the following statistical tool website: PLINK1.9 (https://www.cog-genomics.org/plink/), PLINK2.0 (https://www.cog-genomics.org/plink/2.0/), SAIGE (https://github.com/we izhouUMICH/SAIGE), METAL (https://github.com/statgen/METAL), Fine Mapping (Wakefield Method, https://rdrr.io/cran/gtx/man/abf.Wakefield.html), LDSC (https://github.com/bulik/ldsc), Popcorn (https:// github.com/brielin/Popcorn), LocusZoom (http://locuszoom.org/), FUMA (https://fuma.ctglab.nl/). No custom codes were generated for the present study.
