# Supplementary material for: Genetic study identifies novel genes in developmental dysplasia of the hip
Source: Bone Res. 2026 Mar 31;14:34. doi: 10.1038/s41413-026-00514-8 (PMC13035913; doi:10.1038/s41413-026-00514-8)
Supplement: Supplementary file 1 — Supplementary material [file 41413_2026_514_MOESM1_ESM.docx]

SUPPLEMENTARY MATERIALS

Contents:

Ⅰ. Supplementary Note

Ⅱ. Supplementary figures

1. Supplementary figure 1: Quantile-Quantile plot of each GWAS
2. Supplementary figure 2: Regional plot of Japanese hip dysplasia association results with HLA haplotype
3. Supplementary figure 3: Partitioning heritability enrichment analysis with cell groups and JPN DDH GWAS sumstats
4. Supplementary figure 4: Partitioning heritability enrichment analysis with ATAC data of three cell-types from ChIP atlas and JPN DDH GWAS sumstats
5. Supplementary figure 5: The lead variants overlapped with open chromatin regions in chondrosytes
6. Supplementary figure 6: Comparison of directions of the effect sizes between Japanese DDH and GO hip OA
7. Supplementary figure 7: Principal component analysis of Japanese GWAS

Ⅲ. Supplementary Tables

1. Supplementary table 1: Japanese hip dysplasia association results with HLA haplotype(TOP5)
2. Supplementary table 2: Beta comparison of 3 hip dysplasia lead variants between hip dysplasia and dislocated hip
3. Supplementary table 3: Genetic Correlation between dysplasia and dislocated hip
4. Supplementary table 4: Hip dysplasia and dislocated hip GWAS results of the five significant signals in JPN DDH GWAS
5. Supplementary table 5: Rare variants in JPN DDH GWAS
6. Supplementary table 6: Bayesian fine-mapping results (JPN DDH GWAS)
7. Supplementary table 7: Top 20 GSEA result with the results of JPN DDH by FUMA
8. Supplementary table 8: Partitioning heritability enrichment analysis with cell groups and JPN DDH GWAS sumstats
9. Supplementary table 9: Partitioning heritability enrichment analysis with cell types and JPN DDH GWAS sumstats
10. Supplementary table 10: Partitioning heritability enrichment analysis with ATAC data of three cell-types from ChIP atlas and JPN DDH GWAS sumstats
11. Supplementary table 11: Genetic Correlation with other phenotype
12. Supplementary table 12: Trans ethnic genetic correlation between JPN DDH and UK DDH
13. Supplementary table 13: Lead variants list of meta-analysis between JPN DDH GWAS and UK DDH GWAS
14. Supplementary table 14: Top 30 GSEA result with meta-analysis results of DDH (JPN and UK) by FUMA
15. Supplementary table 15: Association results of meta analysis between JPN DDH or non-DDH hip OA and GO Hip OA
16. Supplementary table 16: Trans ethnic genetic correlation between JPN DDH and GO Hip OA
17. Supplementary table 17: Partitioning heritability enrichment analysis with condition-specific chromatin accessibility data in chondrocytes and JPN DDH GWAS sumstats
18. Supplementary table 18: lncRNAs located in the proximity of DDH GWAS association and regulated by allelic imbalance.
19. Supplementary table 19: Minor allele frequencies of lead variants associated with DDH in the JPN GWAS across EAS and EUR (Non-Finnish) populations.
20. Supplementary table 20: Comparison of LD r^2^ between rs146711505 and EUR proxy variants in JPN and EUR populations.

Ⅳ. References

Ⅰ. Supplementary Note

**Dataset descriptions**

NJR descriptive: The National Joint Registry for England, Wales, Northern Ireland, and the Isle of Man (NJR, http://www.njrcentre.org.uk/)

United Kingdom Household Longitudinal Study (UKHLS): The United Kingdom Household Longitudinal Study, also known as Understanding Society (<https://www.understandingsociety.ac.uk>) is a longitudinal panel survey of 40.000 UK households (England, Scotland, Wales and Northern Ireland) representative of the UK population. Participants are surveyed annually since 2009 and contribute information relating to their socioeconomic circumstances, attitudes, and behaviours via a computer assisted interview. The study includes phenotypical data for a representative sample of participants for a wide range of social and economic indicators as well as a biological sample collection encompassing biometric, physiological, biochemical, and haematological measurements and self-reported medical history and medication use.

**Genotyping and quality control of Japanese GWASs**

Genomic DNA was extracted from peripheral venous blood samples using a standard method. Both case and control samples were genotyped using the Illumina Asian Screening Array.

For the quality control of samples, we removed samples whose sex differed between genotype and clinical data. We excluded both sample that were genetically identical (PI-HAT >0.9). PI_HAT is an index of relatedness between two individuals based on identity by descent implemented in PLINK2.0^1^ (https://www.cog-genomics.org/plink2). To identify population stratification, we conducted principal component analysis (PCA) with four populations from HapMap data as the reference: European (CEU), African (YRI), Japanese (JPT), and Han Chinese (CHB) with SmartPCA (https://github.com/chrchang/eigensoft/wiki/smartpca). We generated a scatterplot using principal components (eigenvectors) 1 and 3 and removed outliers (eigenvector 1 ≤-0.005 and eigenvector 1 ≥0.01) from the East Asian (JPT/CHB) cluster (Supplementary figure 7). We also excluded samples with a genotyping call rate of <98%.

For quality control of genotyped SNPs, variants with SNP call rate <99%, MAF <0.01, and Hardy–Weinberg equilibrium p-value <1.00 × 10^–6^ were removed. as previously reported.^2 3^

**Phasing and genotype imputation of Japanese GWASs**

Pre-phasing and SNP imputation was performed with EAGLE (v2.4.1)^4^ and minimac4 (v1.0.0)^5^, respectively, using the 1000 Genomes Project Phase 3^6^ (1KGP 3 [May 2013 n=2,504]) and 3,256 in-house Japanese whole-genome sequence data obtained from BBJ (JEWEL 3K)^3^. We excluded variants with allele frequency differences greater than 0.03 between the control individuals and the reference panel. After imputation, SNPs with an imputation quality of Rsq >0.3 and MAF >0.005 were included in the subsequent association studies.

**Association analysis of hip dysplasia**

We performed association analyses of autosomes of Hip dysplasia GWAS-1, -2 and -3, independently. We conducted each GWAS by applying a generalized linear mixed model using SAIGE (version 0.35.8.3), which consisted of two steps. In Step 1, we fit a null logistic mixed model using genotype data, and the top 10 principal components (PCs) and sex were incorporated as covariates. In Step 2, the single-variant association tests were performed by using the imputed variant dosages. We then meta-analyzed the three GWAS sets with an inverse variance method under a fixed effect model using METAL software. Regarding X chromosomes, we performed a generalized linear mixed model analysis using SAIGE in males and females separately for each GWAS set, with the top 10 PCs as covariates. We then integrated the results of males and females in each GWAS using METAL.

We estimated confounding biases derived from population stratification and cryptic relatedness using LD score regression using LD scores for the East Asian population.

**Association analysis of Dislocated hip GWAS**

We performed association analyses of Dislocated hip GWAS-1, -2 and -3 in the same way as Hip dysplasia GWAS.

**Association analysis of DDH GWAS**

We performed all DDH GWAS by integrating the results of three Hip dysplasia GWASs and three Dislocated hip GWASs using METAL.

**Whole genome sequence (WGS)-based secondary imputation and association analysis of rare variants**

Using the same method as the genotype imputation of Japanese GWASs, we re-imputed the genotypes of three rare-variant regions with the additional in-house 62 samples, which had both WGS data and Asian Screening Array genotype data. This allowed us to first assess the genotype concordance between the WGS and array genotype data and then to re-evaluate the association signals for each region with the follow-up association analysis (SAIGE and meta-analysis by METAL). The imputation quality scores (Rsq) for the lead variants were high (rs142273463: 0.78; rs147057560: 0.73; rs7168702: 0.75), consistent with the primary imputation (Supplementary Table 5). The genotype concordance for heterozygotes between the WGS and array data was overall 72%, with variation among the variants (100% (2/2 carrier samples) for rs147057560, 75% (3/4 carrier samples) for rs7168702, and 0% (0/1 carrier sample) for rs142273463). The association analysis using the results of the secondary imputation showed that all three variants maintained genome-wide significance (Supplementary table 5).

**Association analysis of Non-DDH OA GWAS**

Different from Hip dysplasia and Dislocated hip GWASs, as the total number of non-DDH OA of each three genotyping batches was too small to conduct GWAS for each, we combined three groups together and performed one GWAS using SAIGE. In step 1, we fit a null logistic mixed model using genotype data, and the top 10 principal components (PCs), three genotyping batches and sex were incorporated as covariates. Step 2 was performed in the same way as Hip dysplasia and Dislocated hip GWASs.

**Genotyping and quality control of UK GWAS**

A total of 834 NJR DDH cases had their DNA genotyped with the Illumina HumanCoreExome-24 BeadChip (Illumina, San Diego, USA). The genotypes were using Illumina Genome Studio Gencall algorithm. Similarly, the UKHLS (control) samples were genotyped using the Illumina HumanCoreExome-12v1-0_A chip, with the same genotype calling algorithm. Plink (version 1.90) was used to perform quality control (QC). Both cases and controls underwent QC as follow: (i) call rate < 98%, (ii) gender discrepancy, (iii) excess heterozygosity (heterozygosity rate ≥ 3 SD), (iv) duplicates and/or related (defined as a PIHAT ≥ 0.9 and/or 0.2), (v) ancestry outliers. Variants underwent quality control as follow: (i) call rate < 98%, (ii) Hardy–Weinberg Equilibrium P-value < 1× 10−4. Before QC, there were 498,017 variants in the genotype data of the case samples and 525,314 variants in the control samples. The case and control data were combined based on the overlapping variants between them. A total of 497,831 overlapping typed variants were included post-QC.

**Genotype imputation of UK GWAS**

Prior to genotype imputation, QC was performed (https://www.strand.org.uk/tools/index.html) in order to compare the genotypes to the reference panel. The following checks were implemented: update strand, position, reference/alternative allele assignment, and remove palindromic SNPs if minor allele frequency (MAF) > 0.4, SNPs with differing alleles to the reference, SNPs with > 0.2 allele frequency difference, SNPs not in reference panel. Subsequently, 383,681 SNPs were included for genotype imputation. Genotype imputation was performed on the Sanger Imputation Server (https://imputation.sanger.ac.uk/) using the Haplotype Reference Consortium reference panel (v1.1).

**Association analyses of UK GWAS**

Genotype dosages were analyzed using SAIGE (version 0.44.6.4) under an additive genetic model. A linear mixed model was employed to account for sample relatedness, and saddle point approximation was used to address case-control imbalance. Sex and the first ten principal components of ancestry were included as covariates to control for potential genomic confounders. The analysis focused on genetic variants with MAF ≥ 0.01, imputation INFO score ≥ 0.3, and minor allele count ≥ 5. Genome-wide significance threshold of p-value < 5×10−8 was considered the threshold for significant single variant associations. The Manhattan and Regional plots of each GWAS were created using the code published in GWASlab (<https://cloufield.github.io/gwaslab/>).

**Overlap of GWAS signals and heritability enrichment in regulatory regions in chondrocytes**

We examined the overlap between our GWAS lead variants and condition-specific expression quantitative trait loci (eQTLs), chromatin accessibility, and 3D chromatin structure using the data from human talar cartilage tissue with no known history of arthritis (Kramer et al.). We identified significant overlaps between two lead variants, rs6088815 and rs2425066 (both in high LD with our *GDF5* lead variant rs143384), and eQTLs, suggesting that DDH risk may be mediated through the regulation of *GDF5* expression in chondrocytes. This finding implies that the *GDF5* contribution to DDH reflects fundamental cartilage biology rather than secondary arthritic changes.

We also conducted stratified LD score regression and assessed heritability enrichment of DDH in these chondrocyte-specific regulatory regions. Our analysis did not show significant heritability enrichment (Supplementary Table 17). The lack of broader heritability enrichment may indicate that DDH’s genetic basis is not limited to chondrocyte-specific regulatory elements, or that it involves tissue-specific mechanisms in hip cartilage not fully captured by the talar cartilage data.

**Identifying potentially casual lncRNAs**

To identify potentially causal lncRNAs in bone, we focused on those located in proximity of our GWAS associations and regulated by allelic imbalance (AI), hypothesizing that such lncRNAs could be causal for their respective associations due to the potential regulation by an eQTL colocalizing with GWAS signals. Using the set of 9,052 lncRNAs (2,440 novel and 6,612 known) quantified in acetabular bone by Abood et al., we identified lncRNAs within a 400-kb window (±200 kb from the lncRNA start site) of each GWAS association. The rationale for this genomic distance, as described by Abood et al., was to extend the window to ensure capture of the majority of cis-eQTL effects. We confirmed the presence of multiple lncRNAs within ±200 kb of our GWAS signals (Supplementary Table 18). While these lncRNAs have not been previously linked to DDH or OA, their data origin, acetabular tissue—the directly affected joint in these conditions—may suggest important regulatory functions of these lncRNAs.

**Investigation of non-replicated Japanese specific loci in trans-ancestry meta-analysis**

The lack of replication or attenuation of Japanese specific lead variants in the trans-ancestry meta-analysis is primarily attributable to population specific genetic architectures, including differences in MAF and LD patterns.

We examined the allele frequencies of the lead variants using the gnomAD browser (https://gnomad.broadinstitute.org/), as shown in Supplementary Table 19.

**MAF-driven attenuation:** For most of the Japanese-specific associations (rs142273463, rs147057560, rs7168702, and rs78572420), the primary reason for lack of associations in UK appears to be very low MAF in the UK cohort. Such large MAF disparities likely resulted in their exclusion during quality control or yielded unstable association estimates. This issue was particularly evident with the chromosome 12 lead variant, rs78572420, which was only present in the UK summary statistics but at a low frequency, and showed conflicting effect directions between cohorts (MAF_UK​=0.012 compared to MAF_JPN​=0.222, β_UK​=−0.04 compared to β_JPN​=0.30).

**LD-driven attenuation:** For the variant on chromosome 6 (rs146711505), our investigation confirmed that the lack of replication is due to a profound difference in LD structure, as the allele frequencies are comparable between the populations (MAF_EAS​=0.276; MAF_EUR​=0.343). We subsequently checked the presence of rs146711505 and the LD discrepancy, finding that rs146711505 was not genotyped on the array used for the UK cohorts and was consequently absent from the UK summary statistics. This was primarily because the variant was an indel and not included in the HRC imputation reference panel. However, we confirmed its presence in the UK Biobank genotype data, where it is recorded as 6:1531009_TTG_T (rsID and position match). To estimate the signal, we identified three high-LD proxy variants in the UK Biobank (r^2^>0.8): rs2317961, rs2816294, and rs7739648 (see Supplementary Table 20 for r^2^ values). Crucially, when we checked the LD of these same proxies in our Japanese reference panel, the r^2^ values with rs146711505 were found to be low (r^2^: 0.28−0.29; Supplementary Table 20).

The substantial difference in r^2^ values confirms that the LD structure surrounding rs146711505 is vastly different between the Japanese and UK populations. This provides a clear example of how population-specific LD structure prevented the same causal signal from being captured across the two cohorts, thereby explaining the attenuation in the meta-analysis for this specific locus.

Ⅱ. Supplementary figures


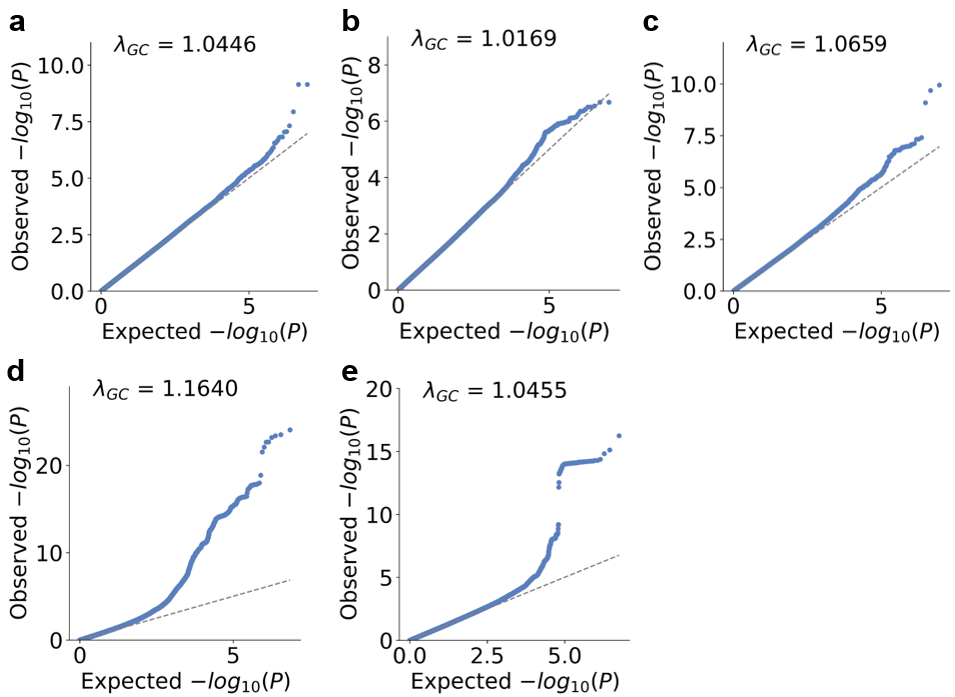


Supplementary figure 1: Quantile-Quantile plot of each GWAS.The x-axis displays the expected –log10 transformed p-values and y-axis displays the observed –log10 transformed p-values.

a: Japanese hip dysplasia GWAS, b: Japanese dislocated hip GWAS, c: Japanese DDH GWAS


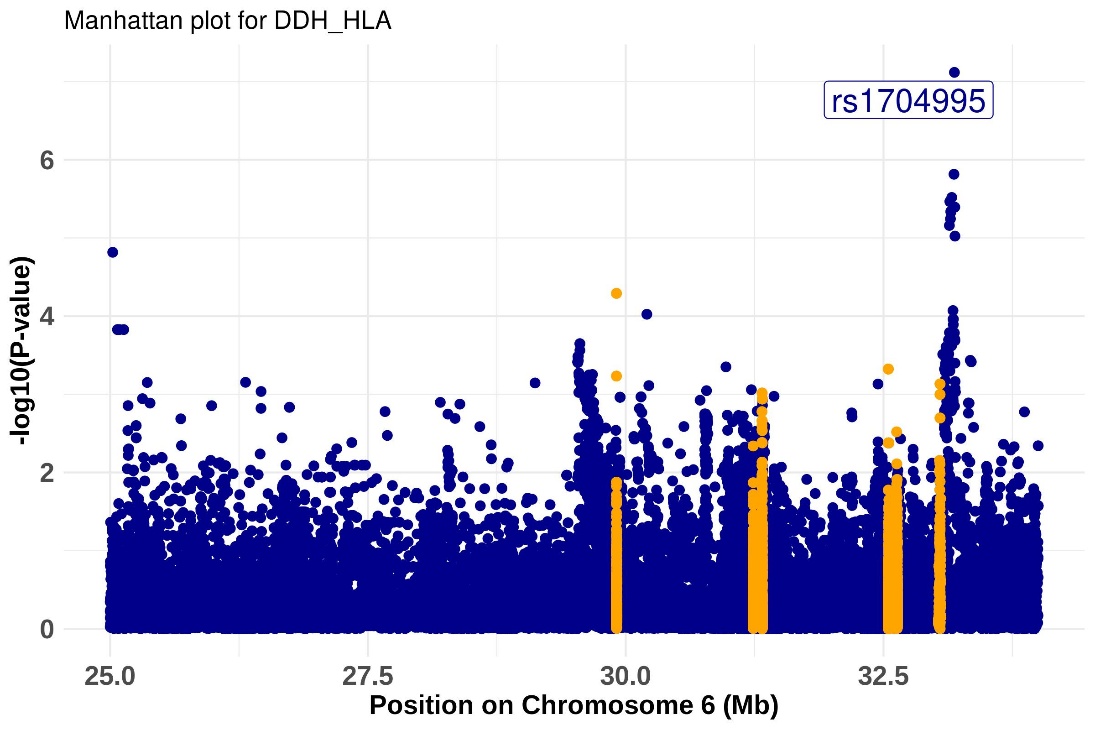
d: Meta-analysis (Japanese DDH and UK DDH), e: Meta-analysis (Japanese DDH/hip OA and GO hip OA).

Supplementary figure 2: A regional plot of association results for Japanese hip dysplasia in the HLA region.

The dots highlighted in yellow represent polymorphisms (SNPs, amino acid residues and HLA alleles) on the HLA genes.


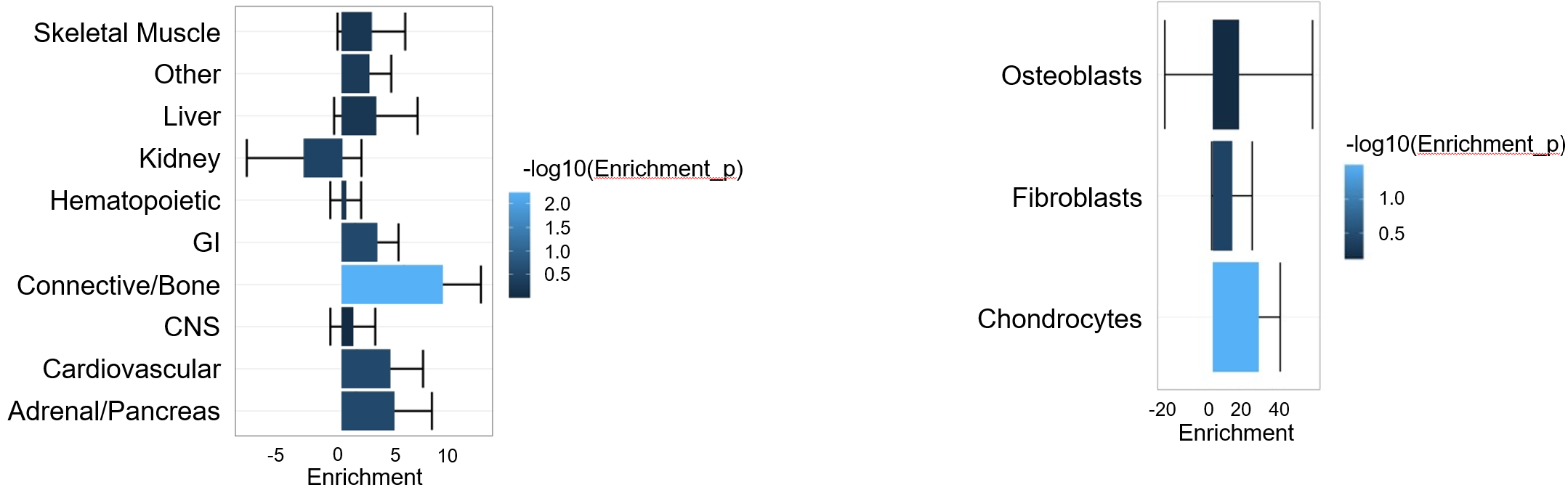


Supplementary figure 3: Partitioning heritability enrichment analysis cell group results (JPN DDH GWAS sumstats). We conducted stratified LD score regression using 220 cell-type-specific annotations of four histone marks (H3K4me1, H3K4me3, H3K9ac, and H3K27ac). The 220 cell-type-specific annotations were divided into 10 cell-type groups (10 in adrenal/pancreas, 34 in central nervous system, 15 in cardiovascular, 6 in connective/bone, 44 in gastrointestinal, 67 in immune/hematopoietic, 5 in kidney, 6 in liver, 10 in skeletal muscle, and 23 in other). We assessed heritability enrichment in histone marks of these ten cell type groups. We excluded variants within the major histocompatibility complex (MHC) region (chromosome 6: 25–34 Mb). We defined significant heritability
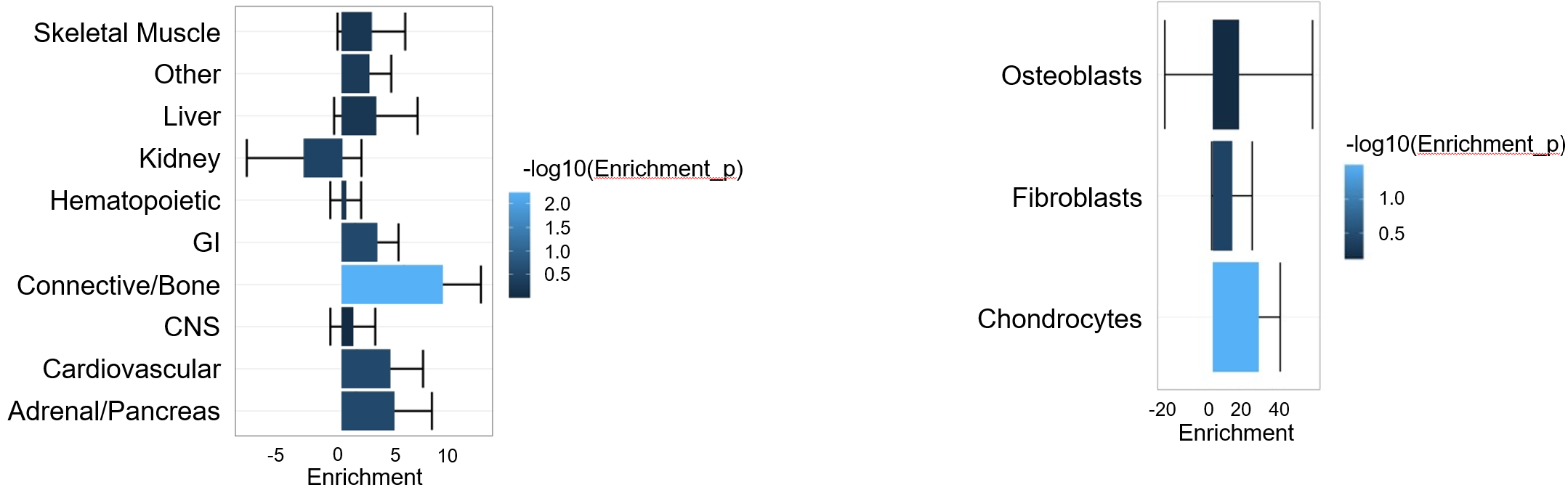
enrichment as those with an FDR < 0.05.

Supplementary figure 4: Partitioning heritability enrichment analysis results (ATAC data from ChIP atlas JPN DDH GWAS sumstats). We performed heritability enrichment analysis using the open chromatin region of three cell-types. We excluded variants within the major histocompatibility complex (MHC) region (chromosome 6: 25–34 Mb). We defined significant heritability enrichment as those with an FDR < 0.05.

Supplementary figure 5: The lead variants overlapped with open chromatin regions in chondrocytes. Above shows rs993471 and rs3753841(r²=0.998). Below shows rs143384 and rs4911494(r²=0.915).


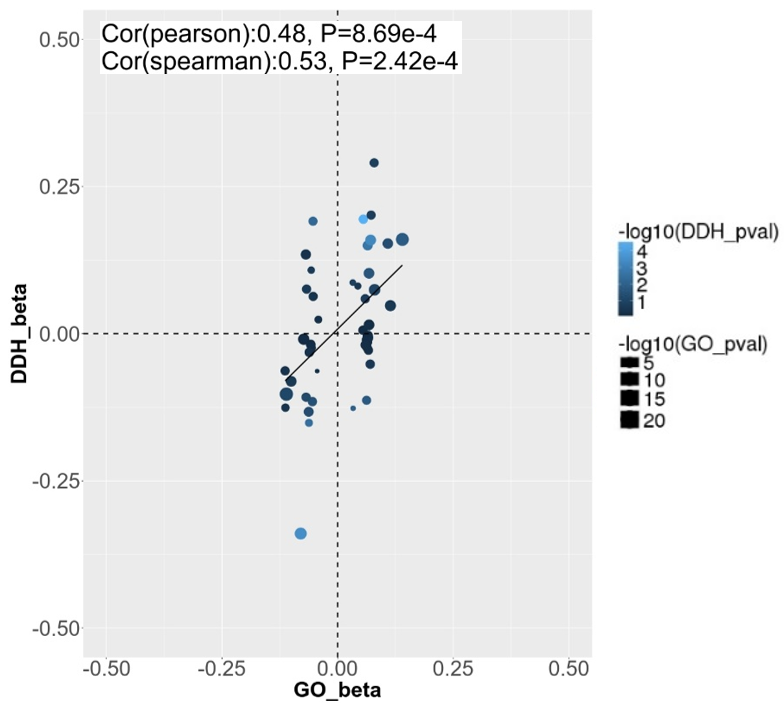
Supplementary figure 6: Comparison of directions of the effect sizes between Japanese DDH and GO hip OA. The dots represent the 45 lead variants identified in the GO hip OA GWAS.


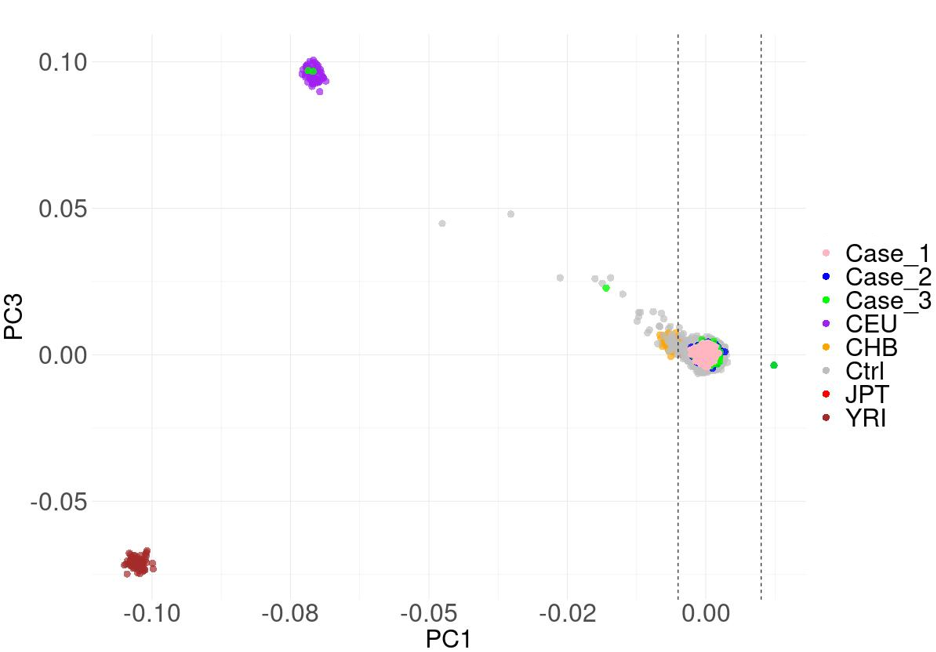


Supplementary figure 7: Principal component analysis of Japanese GWAS. Scatter plots of Principal components (eigenvectors) 1 and PC3. Outliers (eigenvector 1 ≤-0.005 and eigenvector 1 ≥0.01) were removed from the East Asian (JPT/CHB) cluster. Case_1-3, Japanese case (DDH and non DDH OA) set 1-3; YRI, Yoruba in Ibadan (Nigeria); CEU, Utah residents with Northern and Western European ancestry from the CEPH collection; CHB, Han Chinese in Beijing (China); Ctrl, Control; JPT: Japanese in Tokyo (Japan).

Ⅲ. Supplementary tables

Supplementary table 1: Japanese hip dysplasia association results with HLA haplotype. (Top5)

| HLA allele | CHR | POS | Allele1 | Allele2 | Freq1 | OR (95%CI) | P-value | Direction | HetPVal |
| --- | --- | --- | --- | --- | --- | --- | --- | --- | --- |
| HLA-A*33:03:23 | 6 | 29910468 | A | T | 0.9990 | 0.145(0.056-0.377) | 5.1×10^-5^ | --- | 0.84 |
| HLA-DRB1*03 | 6 | 32546552 | A | T | 0.9993 | 0.097(0.027-0.346) | 4.8×10^-4^ | -?- | 0.33 |
| HLA-DRB1*03:01:01:01 | 6 | 32546553 | A | T | 0.9993 | 0.097(0.027-0.346) | 4.8×10^-4^ | -?- | 0.33 |
| HLA-A*11:88 | 6 | 29910341 | A | T | 0.9995 | 0.053(0.011-0.242) | 5.8×10^-4^ | -?- | 0.92 |
| SNPS-DPB1-4815-33048633-exon2-C | 6 | 33048634 | A | T | 0.9311 | 1.522(0.661-3.506) | 7.4×10^-4^ | +++ | 0.55 |

CHR, chromosome; POS, position (position base pair in genome build hg19); Allele1, effect allele; Allele2, alternative allele; Freq1, frequency for effect allele across this analysis; OR, odds ratio; CI, confidence interval; P-value, meta-analysis p-value; Direction, summary of effect direction for each study (Japanese hip dysplasia GWAS set1/Japanese hip dysplasia GWAS set2/Japanese hip dysplasia GWAS set3); HetPVal, p-value for heterogeneity statistic

Supplementary table 2: Beta comparison of 3 hip dysplasia lead variants between hip dysplasia and dislocated hip.

| MarkerName | rsID | Effect Allele | Hip dysplasia | | | Dislocated hip | | | | DDH (meta-analysis) | | | Gene |
| --- | --- | --- | --- | --- | --- | --- | --- | --- | --- | --- | --- | --- | --- |
|  |  |  | Freq | OR(95%CI) | P-value | | Freq | OR(95%CI) | P-value | Freq | OR(95%CI) | P-value |  |
| 6:33187688:T:C | rs1704995 | T | 0.817 | 1.48(1.29-1.71) | 4.8×10^-8^ | | 0.814 | 0.98(0.87-1.11) | 8.7×10^-1^ | 0.816 | 1.31(1.16-1.48) | 6.6×10^-6^ | *COL11A2* |
| 7:71258598:C:T | rs10241320 | T | 0.180 | 1.56(1.35-1.79) | 7.1×10^-10^ | | 0.180 | 0.91(0.80-1.02) | 3.8×10^-1^ | 0.180 | 1.34(1.19-1.51) | 1.8×10^-6^ | *CALN1* |
| 15:50959151:T:C | rs7168702 | T | 0.989 | 0.17(0.09-0.31) | 1.2×10^-8^ | | 0.990 | 0.30(0.26-0.34) | 1.3×10^-2^ | 0.990 | 0.20(0.18-0.23) | 8.1×10^-10^ | *TRPM7* |

Freq, frequency for effect allele; OR; Odds Ratio, DDH; developmental dysplasia of the hip

Supplementary table 3: Genetic Correlation between hip dysplasia and dislocated hip.

|  | rg | se | Z-score | P-value |
| --- | --- | --- | --- | --- |
| JPN hip dysplasia and dislocated hip | 0.996 | 0.590 | 1.700 | 0.091 |

rg, genetic correlation between Japanese hip dysplasia and dislocation; se, standard error

Supplementary table 4: Hip dysplasia and dislocated hip GWAS results of the five significant signals in JPN DDH GWAS.

| rsID | CHR | POS | Gene | Allele1 | Allele2 | Freq1 | OR(95%CI) | P-value | Direction | HetPVal |
| --- | --- | --- | --- | --- | --- | --- | --- | --- | --- | --- |
| Hip dysplasia | |  |  |  |  |  |  |  |  |  |
| rs142273463 | 4 | 177942225 | *VEGF-C* | A | G | 0.995 | 0.157(0.0732-0.338) | 1.9×10^-6^ | --- | 0.96 |
| rs146711505 | 6 | 1531009 | *FOXC1* | T | TTG | 0.236 | 0.752(0.659-0.858) | 2.2×10^-5^ | --- | 0.27 |
| rs147057560 | 9 | 107067481 | *SMC2* | T | G | 0.991 | 0.202(0.110-0.371) | 2.0×10^-7^ | --- | 0.77 |
| rs78572420 | 12 | 47109115 | *SLC38A4* | A | G | 0.774 | 0.742(0.654-0.843) | 4.4×10^-6^ | --- | 0.043 |
| rs7168702 | 15 | 50959151 | *TRPM7* | T | C | 0.989 | 0.170(0.0927-0.312) | 1.2×10^-8^ | --- | 0.69 |
| Dislocated hip | |  |  |  |  |  |  |  |  |  |
| rs142273463 | 4 | 177942225 | *VEGF-C* | A | G | 0.994 | 0.0672(0.0208-0.218) | 6.6×10^-6^ | --- | 0.051 |
| rs146711505 | 6 | 1531009 | *FOXC1* | T | TTG | 0.231 | 0.690(0.556-0.856) | 0.00046 | --- | 0.80 |
| rs147057560 | 9 | 107067481 | *SMC2* | T | G | 0.991 | 0.178(0.0709-0.448) | 0.00025 | --- | 0.097 |
| rs78572420 | 12 | 47109115 | *SLC38A4* | A | G | 0.779 | 0.726(0.585-0.901) | 0.0025 | --- | 0.14 |
| rs7168702 | 15 | 50959151 | *TRPM7* | T | C | 0.990 | 0.298(0.114-0.780) | 0.013 | +-- | 0.11 |

JPN, Japan; CHR, chromosome; POS, position (position base pair in genome build hg19); Allele1, effect allele; Allele2, alternative allele; Freq1, frequency for effect allele across this analysis; OR, odds ratio; CI, confidence interval; P-value, meta-analysis p-value; Direction, summary of effect direction for each study (GWAS set1/ GWAS set2/ GWAS set3); HetPVal, p-value for heterogeneity statistic

Supplementary table 5: Rare variants in JPN DDH GWAS

| rsID | Allele1 | Allele2 | Primary summary statistics | | | | | Secondary summary statistics | | | | | |
| --- | --- | --- | --- | --- | --- | --- | --- | --- | --- | --- | --- | --- | --- |
|  |  |  | Freq1 | Rsq | OR(95%CI) | P-value | Direction | | Freq1 | Rsq | OR(95%CI) | P-value | Direction |
| rs142273463 | A | G | 0.994 | 0.788 | 0.13(0.07-0.24) | 1.83E-10 | ------ | | 0.994 | 0.784 | 0.13(0.07-0.24) | 1.83E-10 | ------ |
| rs147057560 | T | G | 0.991 | 0.729 | 0.20(0.12-0.33) | 5.19E-10 | ------ | | 0.991 | 0.729 | 0.20(0.12-0.33) | 5.19E-10 | ------ |
| rs7168702 | T | C | 0.99 | 0.754 | 0.20(0.12-0.34) | 1.04E-09 | ---+-- | | 0.99 | 0.755 | 0.20(0.12-0.34) | 1.04E-09 | ---+-- |

JPN, Japan; Allele1, effect allele; Allele2, alternative allele; Freq1, frequency for effect allele across this analysis; OR, odds ratio; CI, confidence interval; P-value, meta-analysis p-value; Direction, summary of effect direction for each study (GWAS set1/ GWAS set2/ GWAS set3)

Supplementary table 6: Bayesian fine-mapping results (JPN DDH GWAS)

| Region | Rank | CHR:POS | rsID | PP |
| --- | --- | --- | --- | --- |
| *VEGF-C* | 1 | 4:177942225 | rs142273463 | 0.9997 |
| *FOXC1* | 1 | 6:1531009 | rs146711505 | 0.9604 |
| *SMC2* | 1 | 9:107067481 | rs147057560 | 0.99996 |
| *SLC38A4* | 1 | 12:47109115 | rs78572420 | 0.06978 |
|  | 2 | 12:47106260 | rs2408618 | 0.05913 |
|  | 3 | 12:47079867 | rs759703 | 0.03400 |
|  | 4 | 12:47084530 | rs75530829 | 0.03330 |
|  | 5 | 12:47081894 | rs76649044 | 0.02803 |
|  | 6 | 12:47088715 | rs79517438 | 0.02753 |
|  | 7 | 12:47079647 | rs759702 | 0.02720 |
|  | 8 | 12:47078792 | rs12303838 | 0.02720 |
|  | 9 | 12:47062267 | rs76515116 | 0.02632 |
|  | 10 | 12:47077134, 12:47076186 | rs58190601, rs76462815 | 0.02464 |
|  | 12 | 12:47073558 | rs61562961 | 0.02442 |
|  | 13 | 12:47067649 | rs147834350 | 0.02259 |
|  | 14 | 12:47054654 | rs12296535 | 0.02132 |
|  | 15 | 12:47053646 | rs12321468 | 0.02118 |
|  | 16 | 12:47026773 | rs1118657 | 0.01877 |
|  | 17 | 12:47037499 | rs112539298 | 0.01860 |
|  | 18 | 12:47033479, 12:47037847, 12:47037635, 12:47038550, 12:47043174 | rs57964461, rs73293953, rs12307183, rs11504533, rs12318364 | 0.01844 |
|  | 23 | 12:47027153, 12:47027537 | rs1118658, rs1895088 | 0.01828 |
|  | 25 | 12:47027937 | rs79605106 | 0.01791 |
|  | 26 | 12:47035006 | rs79107816 | 0.01764 |
|  | 27 | 12:47034537 | rs115017684 | 0.01733 |
|  | 28 | 12:47110455, 12:47090986 | rs1937732006, rs73282144 | 0.01267 |
|  | 30 | 12:47028531 | rs58871310 | 0.01264 |
|  | 31 | 12:47006790 | rs12302091 | 0.01219 |
|  | 32 | 12:47091005 | rs7952809 | 0.01215 |
|  | 33 | 12:47090806, 12:47093014 | rs2193651, rs4768745 | 0.01156 |
| *TRPM7* | 1 | 15:50959151 | rs7168702 | 0.9890 |

JPN, Japan; CHR, chromosome; POS, position (position base pair in genome build hg19); DDH, developmental dysplasia of the hip; PP, posterior probability

Supplementary table 7: Top 20 GSEA result with the results of JPN DDH by FUMA.

| PATHWAY | NGENES | BETA | BETA_STD | SE | P |
| --- | --- | --- | --- | --- | --- |
| GOMF_ALDEHYDE_DEHYDROGENASE_NAD_P_PLUS_ACTIVITY | 17 | 0.90 | 0.027 | 0.20 | 4.2×10^-6^ |
| PID_AP1_PATHWAY | 69 | 0.42 | 0.025 | 0.10 | 1.9×10^-5^ |
| GOBP_REGULATION_OF_CELL_MIGRATION_INVOLVED_IN_SPROUTING_ANGIOGENESIS | 37 | 0.58 | 0.026 | 0.14 | 1.9×10^-5^ |
| GOBP_POSITIVE_REGULATION_OF_CELL_MIGRATION_INVOLVED_IN_SPROUTING_  ANGIOGENESIS | 20 | 0.77 | 0.025 | 0.19 | 4.0×10^-5^ |
| GOCC_COLLAGEN_TYPE_XI_TRIMER | 5 | 1.8 | 0.029 | 0.45 | 4.2×10^-5^ |
| GOMF_PHOSPHOPROTEIN_BINDING | 87 | 0.34 | 0.023 | 0.087 | 4.5×10^-5^ |
| GOBP_RHYTHMIC_BEHAVIOR | 46 | 0.52 | 0.025 | 0.13 | 5.2×10^-5^ |
| GOBP_RESPONSE_TO_PURINE_CONTAINING_COMPOUND | 136 | 0.27 | 0.023 | 0.070 | 5.6×10^-5^ |
| MARIADASON_RESPONSE_TO_BUTYRATE_SULINDAC_4 | 15 | 0.70 | 0.020 | 0.19 | 9.2×10^-5^ |
| GOBP_ENDODERM_FORMATION | 54 | 0.44 | 0.024 | 0.12 | 9.3×10^-5^ |
| GOBP_ENDODERMAL_CELL_DIFFERENTIATION | 46 | 0.46 | 0.023 | 0.13 | 0.00010 |
| REACTOME_ACTIVATION_OF_NIMA_KINASES_NEK9_NEK6_NEK7 | 7 | 1.4 | 0.028 | 0.40 | 0.00012 |
| GOBP_NEGATIVE_REGULATION_OF_NF_KAPPAB_TRANSCRIPTION_FACTOR_ACTIVITY | 79 | 0.32 | 0.021 | 0.090 | 0.00014 |
| GOBP_RESPONSE_TO_ORGANOPHOSPHORUS | 122 | 0.27 | 0.021 | 0.075 | 0.00017 |
| GOCC_FIBRILLAR_COLLAGEN_TRIMER | 12 | 1.02 | 0.026 | 0.29 | 0.00017 |
| REACTOME_NUCLEAR_ENVELOPE_BREAKDOWN | 51 | 0.43 | 0.022 | 0.12 | 0.00018 |
| GOBP_VITAMIN_METABOLIC_PROCESS | 102 | 0.27 | 0.019 | 0.078 | 0.00031 |
| CROONQUIST_NRAS_SIGNALING_DN | 70 | 0.32 | 0.019 | 0.094 | 0.00033 |
| LEE_LIVER_CANCER_E2F1_UP | 61 | 0.39 | 0.022 | 0.11 | 0.00034 |
| MARKEY_RB1_CHRONIC_LOF_UP | 105 | 0.26 | 0.019 | 0.077 | 0.00036 |

This gene-set enrichment analysis was adjusted for multiple testing using the FDR and threshold is FDR < 0.05. JPN, Japan; DDH, developmental dysplasia of the hip; NGENES, number of genes; BETA_STD, beta standard deviation; SE, standard error

Supplementary table 8: Partitioning heritability enrichment analysis with cell groups and JPN DDH GWAS sumstats

| cell_group | Enrichment | Enrichment_se | Enrichment_p |
| --- | --- | --- | --- |
| Connective_Bone | 8.7 | 3.3 | 0.0058 |
| Adrenal_Pancreas | 4.5 | 3.3 | 0.27 |
| Cardiovascular | 4.2 | 2.9 | 0.27 |
| GI | 3.02 | 1.9 | 0.28 |
| Kidney | -3.3 | 5.0 | 0.35 |
| Other | 2.3 | 1.9 | 0.50 |
| Liver | 2.9 | 3.6 | 0.59 |
| Hematopoietic | 0.32 | 1.3 | 0.60 |
| SkeletalMuscle | 2.6 | 2.9 | 0.60 |
| CNS | 0.94 | 1.9 | 0.98 |

JPN, Japan; DDH, developmental dysplasia of the hip; se, standard error; GI, gastrointestinal, CNS; central nervous system

Supplementary table 9: Partitioning heritability enrichment analysis with cell types and JPN DDH GWAS sumstats

| Cell | mark | Enrichment | Enrichment_SE | Enrichment_p | Coefficient | Coefficient_SE | Coefficient_z.score | FDR |
| --- | --- | --- | --- | --- | --- | --- | --- | --- |
| Chondrogenic_differentiation | H3K27ac | 26.9 | 9.2 | 4.7×10^-5^ | 2.5×10^-7^ | 7.2×10^-8^ | 3.4 | 0.010 |
| CD8_naive_primary_(UCSF-UBC) | H3K4me1 | -15.4 | 6.2 | 9.5×10^-5^ | -2.5×10^-7^ | 6.4×10^-8^ | -4.0 | 0.010 |
| CD4_naive_primary | H3K4me3 | -35.5 | 15.01 | 0.000364 | -5.9×10^-7^ | 1.6×10^-7^ | -3.6 | 0.027 |
| CD4+_CD25int_CD127+_Tmem_primary | H3K4me3 | -40.9 | 17.6 | 0.000608 | -6.6×10^-7^ | 2.1×10^-7^ | -3.2 | 0.033 |
| CD8_naive_primary_(BI) | H3K4me3 | -36.5 | 16.2 | 0.00104 | -5.8×10^-7^ | 1.8×10^-7^ | -3.2 | 0.038 |
| CD8_naive_primary_(BI) | H3K4me1 | -10.6 | 5.02 | 0.00115 | -1.9×10^-7^ | 5.5×10^-8^ | -3.4 | 0.038 |
| CD4+_CD25-_Th_primary | H3K4me3 | -33.03 | 15.04 | 0.00136 | -5.4×10^-7^ | 1.8×10^-7^ | -3.07 | 0.038 |
| CD4_memory_primary | H3K4me3 | -31.5 | 14.4 | 0.00164 | -5.2×10^-7^ | 1.6×10^-7^ | -3.1 | 0.038 |
| CD4+_CD25-_CD45RA+_naive_primary | H3K4me3 | -30.7 | 14.2 | 0.00171 | -4.9×10^-7^ | 1.6×10^-7^ | -3.02 | 0.038 |
| CD25-_IL17-_Th_stim_MACS | H3K27ac | -10.9 | 5.4 | 0.00175 | -1.7×10^-7^ | 5.2×10^-8^ | -3.2 | 0.038 |
| CD25-_CD45RA+_naive | H3K27ac | -11.9 | 5.7 | 0.00250 | -1.8×10^-7^ | 5.6×10^-8^ | -3.2 | 0.050 |
| CD4_primary | H3K4me3 | -32.3 | 15.3 | 0.00281 | -5.3×10^-7^ | 1.8×10^-7^ | -2.9 | 0.051 |
| CD3_primary_(UW) | H3K4me3 | -35.3 | 16.9 | 0.00341 | -5.5×10^-7^ | 2.0×10^-7^ | -2.8 | 0.058 |
| CD8_memory_primary | H3K4me3 | -32.3 | 15.2 | 0.00425 | -5.0×10^-7^ | 1.8×10^-7^ | -2.8 | 0.064 |
| Osteoblast | H3K27ac | 15.8 | 6.7 | 0.00436 | 1.2×10^-7^ | 6.3×10^-8^ | 1.8 | 0.064 |
| CD25-_IL17+_Th17_stim | H3K27ac | -14.0 | 7.2 | 0.00518 | -2.0×10^-7^ | 6.9×10^-8^ | -2.9 | 0.068 |
| CD4+_CD25+_CD127-_Treg_primary | H3K4me3 | -28.6 | 14.2 | 0.00522 | -4.7×10^-7^ | 1.7×10^-7^ | -2.7 | 0.068 |
| CD15_primary | H3K4me1 | -9.4 | 5.03 | 0.00559 | -1.6×10^-7^ | 4.9×10^-8^ | -3.3 | 0.068 |
| CD8_memory_primary | H3K4me1 | -9.1 | 4.8 | 0.00593 | -1.6×10^-7^ | 5.8×10^-8^ | -2.8 | 0.069 |
| CD4_naive_primary | H3K4me1 | -8.6 | 4.6 | 0.00738 | -1.5×10^-7^ | 5.6×10^-8^ | -2.7 | 0.081 |
| Pancreatic_islets | H3K9ac | -51.5 | 24.2 | 0.00886 | -6.8×10^-7^ | 2.9×10^-7^ | -2.3 | 0.088 |
| Treg_primary | H3K4me3 | -17.1 | 8.79 | 0.00892 | -2.7×10^-7^ | 1.01×10^-7^ | -2.6 | 0.088 |
| CD4+_CD25-_IL17+_PMA_Ionomycin_stim_Th17_primary | H3K4me3 | -27.4 | 14.01 | 0.00921 | -4.3×10^-7^ | 1.7×10^-7^ | -2.5 | 0.088 |
| Peripheralblood_mononuclear_primary | H3K4me1 | -35.6 | 17.7 | 0.00990 | -5.0×10^-7^ | 2.1×10^-7^ | -2.3 | 0.090 |
| CD3_primary_(UW) | H3K4me1 | -8.8 | 5.1 | 0.0104 | -1.6×10^-7^ | 5.6×10^-8^ | -2.8 | 0.090 |
| Kidney | H3K9ac | -29.2 | 15.4 | 0.0111 | -4.2×10^-7^ | 1.9×10^-7^ | -2.2 | 0.090 |
| CD3_primary_(BI) | H3K4me3 | -25.5 | 13.7 | 0.0116 | -4.0×10^-7^ | 1.5×10^-7^ | -2.6 | 0.090 |
| Substantia_nigra | H3K4me3 | -30.9 | 16.8 | 0.0118 | -4.7×10^-7^ | 1.8×10^-7^ | -2.6 | 0.090 |
| CD4+_CD25-_Th_primary | H3K4me1 | -6.8 | 4.0 | 0.0119 | -1.3×10^-7^ | 4.9×10^-8^ | -2.7 | 0.090 |
| CD4+_CD25+_CD127-_Treg_primary | H3K4me1 | -10.01 | 5.7 | 0.0126 | -1.7×10^-7^ | 6.7×10^-8^ | -2.5 | 0.092 |
| CD4+_CD25-_IL17-_PMA_Ionomycin_stim_MACS_Th_sprimary | H3K4me3 | -18.06 | 9.8 | 0.0129 | -3.1×10^-7^ | 1.2×10^-7^ | -2.7 | 0.092 |
| Peripheralblood_mononuclear_primary | H3K4me3 | -34.98 | 18.6 | 0.0141 | -5.0×10^-7^ | 2.4×10^-7^ | -2.1 | 0.095 |
| CD3_primary_(BI) | H3K4me1 | -9.23 | 5.2 | 0.0142 | -1.6×10^-7^ | 6.1×10^-8^ | -2.6 | 0.095 |
| CD3_primary | H3K27ac | -8.97 | 5.2 | 0.0151 | -1.6×10^-7^ | 5.5×10^-8^ | -2.9 | 0.098 |
| CD15_primary | H3K4me3 | -22.83 | 12.2 | 0.0162 | -3.1×10^-7^ | 1.4×10^-7^ | -2.3 | 0.1 |
| Rectal_mucosa | H3K9ac | -21.19 | 11.7 | 0.0163 | -3.4×10^-7^ | 1.3×10^-7^ | -2.5 | 0.1 |

This analysis was adjusted for multiple testing using the FDR. Cell types whose FDR is < 0.1 are discribed. JPN, Japan; DDH, developmental dysplasia of the hip; SE, standard error

Supplementary table 10: Partitioning heritability enrichment analysis with ATAC data of three cell-types from ChIP atlas and JPN DDH GWAS sumstats

| cell_group | Enrichment | Enrichment_se | Enrichment_p |
| --- | --- | --- | --- |
| Chondrocytes | 24.1 | 11.5 | 0.032 |
| Fibroblasts | 10.0 | 10.7 | 0.39 |
| Osteoblasts | 13.6 | 39.2 | 0.75 |

DDH, developmental dysplasia of the hip; se, standard error

Supplementary table 11: Genetic Correlation with other phenotype.

| Trait | rg | se | z | P |
| --- | --- | --- | --- | --- |
| Keloid | 0.74 | 0.27 | 2.79 | 0.00530 |
| CHC | -0.62 | 0.25 | -2.5 | 0.0119 |
| CeCa | -0.53 | 0.32 | -1.65 | 0.0987 |
| Glaucoma | -0.31 | 0.19 | -1.60 | 0.108 |
| EnCa | -0.46 | 0.39 | -1.18 | 0.238 |
| Epilepsy | -0.71 | 0.60 | -1.17 | 0.243 |
| T2D | -0.10 | 0.088 | -1.16 | 0.248 |
| Asthma | -0.17 | 0.15 | -1.11 | 0.267 |
| NS | 0.46 | 0.42 | 1.10 | 0.271 |
| LuCa | -0.32 | 0.31 | -1.03 | 0.303 |
| HepCa | -0.90 | 0.93 | -0.97 | 0.331 |
| CHB | 0.27 | 0.28 | 0.96 | 0.335 |
| PaCa | -0.65 | 0.69 | -0.95 | 0.345 |
| MI | -0.096 | 0.11 | -0.90 | 0.366 |
| CHF | -0.17 | 0.20 | -0.83 | 0.407 |
| AD | -0.18 | 0.23 | -0.78 | 0.434 |
| BrCa | 0.12 | 0.20 | 0.58 | 0.562 |
| GaCa | 0.10 | 0.18 | 0.55 | 0.582 |
| OPLL | 0.086 | 0.16 | 0.53 | 0.597 |
| Knee_OA | -0.16 | 0.31 | -0.53 | 0.600 |
| CAD | -0.048 | 0.095 | -0.51 | 0.611 |
| RA | 0.079 | 0.16 | 0.50 | 0.618 |
| CeAn | 0.078 | 0.17 | 0.45 | 0.651 |
| AIS | 0.042 | 0.10 | 0.41 | 0.682 |
| EsCa | 0.078 | 0.19 | 0.41 | 0.682 |
| OvCa | 0.26 | 0.64 | 0.41 | 0.685 |
| UF | -0.082 | 0.23 | -0.36 | 0.723 |
| PrCa | -0.045 | 0.13 | -0.34 | 0.733 |
| Cataract | -0.10 | 0.29 | -0.34 | 0.736 |
| GD | -0.037 | 0.17 | -0.22 | 0.829 |
| Urolithiasis | 0.034 | 0.16 | 0.21 | 0.836 |
| SAP | -0.025 | 0.13 | -0.19 | 0.851 |
| Osteoporosis | -0.034 | 0.21 | -0.16 | 0.875 |
| CoCa | 0.027 | 0.20 | 0.14 | 0.893 |
| PTB | 0.045 | 0.42 | 0.11 | 0.914 |
| IS | 0.018 | 0.19 | 0.097 | 0.923 |
| PAD | -0.015 | 0.16 | -0.092 | 0.927 |
| Cirrhosis | -0.037 | 0.41 | -0.091 | 0.927 |
| AF | -0.011 | 0.12 | -0.086 | 0.931 |
| UAP | -0.010 | 0.17 | -0.058 | 0.954 |
| COPD | 0.0095 | 0.20 | 0.049 | 0.961 |
| Pollinosis | -0.010 | 0.22 | -0.047 | 0.962 |

rg, genetic correlation between Japanese hip dysplasia and dislocation; se, standard error; z, z-score; P, p-value; CHC, Chronic hepatitis C; CeCa, Cervical cancer; EnCa, Endometrial cancer; T2D, Type 2 diabetes; NS, Nephrotic syndrome; LuCa, Lung cancer; HepCa, Hepatocellular carcinoma; CHB, Chronic hepatitis B; PaCa, Pancreatic cancer; MI, myocardial infarction; CHF, Congestive heart failure; AD, Atopic dermatitis; BrCa, Breast cancer; GaCa, Gastric cancer; OPLL, ossification of the posterior longitudinal ligament; Knee_OA, knee osteoarthritis; CAD, Coronary artery disease; RA, Rheumatoid arthritis; CeAn, Cerebral aneurysm; AIS, adolescent idiopathic scoliosis; EsCa, Esophageal cancer; OvCa, Ovarian cancer; UF, Uterine fibroids; PrCa, Prostate cancer; GD, Graves' disease; SAP, severe acute pancreatitis; CoCa, Colorectal cancer; PTB, Pulmonary tuberculosis; IS, Ischemic stroke; PAD, Peripheral artery disease; AF, atrial fibrillation; UAP, unstable angina pectoris; COPD, Chronic obstructive pulmonary disease; Pollinosis,

Supplementary table 12: Trans ethnic genetic correlation between JPN DDH and UK DDH.

| Genetic correlation between JPN DDH and UK DDH calculated by popcorn* | | | | |
| --- | --- | --- | --- | --- |
| Val | (obs) | SE | Z | P |
| UK h1^2 | 0.0804 | 0.1414 | 0.5689 | 0.5694 |
| JPN h2^2 | 0.0334 | 0.0146 | 2.2879 | 0.0221 |
| pge | 0.999996 | 0.000377 | 0.01066 | 0.99149 |

JPN, Japan; DDH, developmental dysplasia of the hip; obs, the common-SNP observed scale heritability for both populations (UK and JPN); pge, the genetic effect correlation; SE, standerd error; Z, Z-score; P, p-value

Supplementary table 13: Lead variants list of meta-analysis between JPN DDH GWAS and UK DDH GWAS.

| JPN DDH | | | | | | | | |  |
| --- | --- | --- | --- | --- | --- | --- | --- | --- | --- |
| MarkerName | rsID | A1 | A2 | Freq1 | INFO | OR(95%CI) | P-value | Number |  |
| 1:103385373 | rs993471 | A | G | 0.65 | 0.964 | 1.23(1.12-1.36) | 2.5×10^-5^ | 25085 |  |
| 20:34025756 | rs143384 | A | G | 0.77 | 0.999 | 1.28(1.16-1.43) | 5.9×10^-6^ | 25085 |  |
| UK DDH | | | | | | | | |  |
| MarkerName | rsID | A1 | A2 | Freq1 | INFO | OR(95%CI) | P-value | Number |  |
| 1:103385373 | rs993471 | A | G | 0.62 | 0.999 | 1.23(1.10-1.38) | 2.4×10^-4^ | 10735 |  |
| 20:34025756 | rs143384 | G | A | 0.41 | 1 | 0.657(0.588-0.735) | 1.7×10^-13^ | 10735 |  |
| Meta | | | | | | | | | |
| MarkerName | rsID | A1 | A2 | Freq1 | FreqSE | OR(95%CI) | P-value | Number | Direction |
| 1:103385373 | rs993471 | A | G | 0.64 | 0.017 | 1.23(1.15-1.33) | 2.2×10^-8^ | 35820 | +++++++ |
| 20:34025756 | rs143384 | A | G | 0.69 | 0.090 | 1.39(1.29-1.50) | 5.8×10^-17^ | 35820 | +++++++ |

JPN, Japan; DDH, developmental dysplasia of the hip; GWAS, genome-wide association studies; A1, effect allele; A2, alternative allele; Freq1, frequency for effect allele across this analysis; INFO, Imputation score; OR, odds ratio; CI, confidence interval; Direction, summary of effect direction for each study(UK-DDH GWAS /Japanese hip dysplasia GWAS set1/Japanese hip dysplasia GWAS set2/Japanese hip dysplasia GWAS set3/Japanese dislocated hip GWAS set1/Japanese dislocated hip GWAS set2/Japanese dislocated hip GWAS set3)

Supplementary table 14: Top 30 GSEA result with meta-analysis results of DDH (JPN and UK) by FUMA.

| PATHWAY | NGENES | BETA | BETA_STD | SE | P |
| --- | --- | --- | --- | --- | --- |
| NIKOLSKY_BREAST_CANCER_20Q11_AMPLICON | 31 | 1.68 | 0.0687 | 0.311 | 3.08×10^-8^ |
| GOCC_COLLAGEN_TYPE_XI_TRIMER | 5 | 1.94 | 0.0318 | 0.405 | 8.30×10^-7^ |
| WU_HBX_TARGETS_3_UP | 18 | 0.921 | 0.0286 | 0.193 | 8.99×10^-7^ |
| DAVICIONI_RHABDOMYOSARCOMA_PAX_FOXO1_FUSION_DN | 14 | 0.997 | 0.0273 | 0.243 | 2.10×10^-5^ |
| GOBP_EMBRYONIC_SKELETAL_SYSTEM_MORPHOGENESIS | 90 | 0.418 | 0.0290 | 0.103 | 2.65×10^-5^ |
| GOBP_PENETRATION_OF_ZONA_PELLUCIDA | 9 | 1.2798 | 0.0281 | 0.317 | 2.74×10^-5^ |
| GOBP_EMBRYONIC_CRANIAL_SKELETON_MORPHOGENESIS | 42 | 0.565 | 0.0268 | 0.142 | 3.57×10^-5^ |
| JOHNSTONE_PARVB_TARGETS_1_UP | 6 | 1.26 | 0.0226 | 0.343 | 0.000122 |
| GOBP_REGULATION_OF_SPINDLE_ORGANIZATION | 47 | 0.446 | 0.0224 | 0.124 | 0.000159 |
| REACTOME_CYCLIN_D_ASSOCIATED_EVENTS_IN_G1 | 45 | 0.440 | 0.0216 | 0.122 | 0.000162 |
| GOBP_SUBSTANTIA_NIGRA_DEVELOPMENT | 40 | 0.408 | 0.0189 | 0.114 | 0.000177 |
| STANELLE_E2F1_TARGETS | 26 | 0.554 | 0.0207 | 0.156 | 0.000187 |
| GOBP_EMBRYONIC_SKELETAL_SYSTEM_DEVELOPMENT | 123 | 0.315 | 0.0255 | 0.0886 | 0.00019 |
| GOCC_RNA_CAP_BINDING_COMPLEX | 10 | 0.853 | 0.0198 | 0.242 | 0.000215 |
| GOBP_ACROSOMAL_VESICLE_EXOCYTOSIS | 9 | 1.04 | 0.0228 | 0.298 | 0.000254 |
| ONDER_CDH1_TARGETS_3_UP | 15 | 0.807 | 0.0229 | 0.238 | 0.000351 |
| GOBP_NEUROENDOCRINE_CELL_DIFFERENTIATION | 15 | 0.762 | 0.0216 | 0.225 | 0.000361 |
| GOBP_REGULATION_OF_CELL_DIVISION | 172 | 0.223 | 0.0213 | 0.0660 | 0.000363 |
| REACTOME_FGFRL1_MODULATION_OF_FGFR1_SIGNALING | 13 | 0.822 | 0.0217 | 0.244 | 0.000371 |
| GOMF_GTPASE_INHIBITOR_ACTIVITY | 12 | 0.866 | 0.0220 | 0.257 | 0.000371 |
| REACTOME_ONCOGENE_INDUCED_SENESCENCE | 32 | 0.487 | 0.0202 | 0.145 | 0.000376 |
| REACTOME_UPTAKE_OF_DIETARY_COBALAMINS_INTO_ENTEROCYTES | 10 | 0.967 | 0.0224 | 0.288 | 0.000393 |
| GOBP_UDP_N_ACETYLGLUCOSAMINE_BIOSYNTHETIC_PROCESS | 9 | 1.32 | 0.0291 | 0.397 | 0.000422 |
| GOMF_RNA_7_METHYLGUANOSINE_CAP_BINDING | 12 | 0.815 | 0.0207 | 0.245 | 0.000452 |
| REACTOME_DISEASES_OF_GLYCOSYLATION | 132 | 0.248 | 0.0208 | 0.0756 | 0.000513 |
| GOBP_REGULATION_OF_SPINDLE_ASSEMBLY | 33 | 0.494 | 0.0208 | 0.151 | 0.000532 |
| GOBP_THYROID_STIMULATING_HORMONE_SECRETING_CELL_DIFFERENTIATION | 6 | 0.977 | 0.0175 | 0.299 | 0.000549 |
| REACTOME_SIGNALING_BY_FGFR2_IIIA_TM | 19 | 0.556 | 0.0178 | 0.171 | 0.000565 |
| BIOCARTA_FBW7_PATHWAY | 9 | 0.949 | 0.0209 | 0.292 | 0.000585 |
| GOBP_REGULATION_OF_BONE_DEVELOPMENT | 8 | 1.13 | 0.0234 | 0.348 | 0.000592 |

This gene-set enrichment analysis(GSEA) was adjusted for multiple testing using the FDR and threshold is FDR < 0.05. DDH, developmental dysplasia of the hip; NGENES, number of genes; BETA_STD, beta standard deviation; SE, standard error

Supplementary table 15: Association results of meta analysis between JPN DDH or non-DDH hip OA and GO Hip OA

| rsID | CHR | POS | Allele1 | Allele2 | Freq1 | OR(95%CI) | P-value | Direction | HetPVal | Novel |
| --- | --- | --- | --- | --- | --- | --- | --- | --- | --- | --- |
| rs11164653 | 1 | 103464210 | T | C | 0.410 | 0.921(0.905-0.937) | 1.4×10^-19^ | ----+--- | 0.13 |  |
| rs4411121 | 1 | 118757034 | T | C | 0.327 | 1.07(1.05-1.09) | 1.0×10^-12^ | +++++++- | 0.57 |  |
| rs67924081 | 11 | 65342981 | A | G | 0.746 | 1.06(1.04-1.09) | 1.5×10^-9^ | ++---+-+ | 0.42 |  |
| rs61734601 | 11 | 67184725 | A | G | 0.0788 | 0.904(0.873-0.936) | 1.1×10^-8^ | ---+-++- | 0.16 |  |
| rs1046934 | 1 | 184023529 | A | C | 0.646 | 1.08(1.06-1.10) | 4.5×10^-16^ | +-++++++ | 0.12 |  |
| rs10831477 | 11 | 95797111 | T | G | 0.805 | 1.07(1.04-1.09) | 1.2×10^-8^ | +--+---+ | 0.29 |  |
| rs1809889 | 12 | 124801226 | T | C | 0.285 | 1.06(1.04-1.08) | 1.8×10^-9^ | +--++++- | 0.64 |  |
| rs2605098 | 1 | 219643649 | A | G | 0.333 | 1.07(1.05-1.08) | 1.6×10^-11^ | ++-+---- | 0.94 |  |
| rs10843013 | 12 | 28025196 | A | C | 0.785 | 0.896(0.877-0.915) | 8.6×10^-25^ | ---+---+ | 0.72 |  |
| rs2408618 | 12 | 47106260 | A | T | 0.221 | 1.34(1.21-1.49) | 3.0×10^-8^ | ++++++++ | 0.15 | novel |
| rs79056043 | 12 | 59289598 | A | G | 0.935 | 0.894(0.863-0.927) | 1.3×10^-9^ | -+--+-+- | 0.27 |  |
| rs2242379 | 15 | 63127774 | A | G | 0.333 | 0.947(0.929-0.965) | 2.1×10^-8^ | ---+++-+ | 0.19 |  |
| rs12908498 | 15 | 67366488 | C | G | 0.531 | 1.08(1.06-1.10) | 3.9×10^-17^ | +--+++++ | 0.33 |  |
| rs9940278 | 16 | 53800200 | T | C | 0.430 | 1.06(1.04-1.08) | 2.4×10^-10^ | ++++-+++ | 0.87 |  |
| rs1401796 | 17 | 54839759 | A | C | 0.520 | 0.944(0.928-0.961) | 1.5×10^-10^ | -+-+-++- | 0.26 |  |
| rs4968575 | 17 | 59667116 | C | G | 0.439 | 0.951(0.935-0.968) | 1.9×10^-8^ | --+---+- | 0.42 |  |
| rs2521348 | 17 | 67499717 | T | C | 0.393 | 1.06(1.04-1.08) | 1.1×10^-11^ | ++++++++ | 0.13 |  |
| rs79723785 | 19 | 55818225 | T | C | 0.978 | 0.821(0.770-0.875) | 1.5×10^-9^ | -+-+++-+ | 0.87 |  |
| rs66989638 | 2 | 106689736 | A | G | 0.132 | 1.08(1.06-1.11) | 2.8×10^-9^ | ++++--++ | 0.49 |  |
| rs9976458 | 21 | 40019473 | T | G | 0.234 | 1.07(1.04-1.09) | 3.0×10^-9^ | +++++-++ | 0.27 |  |
| rs8140207 | 22 | 38130459 | T | G | 0.295 | 1.06(1.04-1.08) | 4.9×10^-10^ | +++--+++ | 0.78 |  |
| rs876531 | 2 | 242500662 | T | C | 0.453 | 0.950(0.934-0.967) | 1.9×10^-8^ | -----++- | 0.28 | novel |
| rs2167973 | 2 | 33555177 | A | C | 0.875 | 0.926(0.901-0.951) | 2.7×10^-8^ | ----+-++ | 0.53 | novel |
| rs2862851 | 2 | 70712802 | T | C | 0.462 | 1.06(1.05-1.08) | 1.2×10^-12^ | +++-++-+ | 0.62 |  |
| rs2276749 | 3 | 11643465 | T | C | 0.0566 | 0.887(0.853-0.922) | 1.7×10^-9^ | -----+-+ | 0.014 |  |
| rs9835230 | 3 | 189735461 | A | G | 0.241 | 1.06(1.04-1.09) | 3.2×10^-9^ | +----++- | 0.25 |  |
| rs2268023 | 3 | 52819327 | A | T | 0.414 | 1.07(1.05-1.09) | 2.2×10^-13^ | ++-+-+-+ | 0.69 |  |
| rs1519038 | 3 | 56066102 | T | C | 0.697 | 0.939(0.919-0.960) | 2.6×10^-8^ | -+----+- | 0.19 | novel |
| rs6855246 | 4 | 103112470 | A | G | 0.928 | 0.896(0.864-0.930) | 6.4×10^-^9 | -+--+++- | 0.11 |  |
| rs1913707 | 4 | 13039440 | A | G | 0.603 | 1.07(1.05-1.09) | 1.9×10^-14^ | ++++++++ | 0.98 |  |
| rs59163323 | 4 | 1763318 | A | G | 0.200 | 0.929(0.908-0.951) | 5.2×10^-10^ | -+---+-+ | 0.60 |  |
| rs788857 | 4 | 82138551 | A | G | 0.703 | 0.947(0.929-0.965) | 2.4×10^-8^ | --+-+--+ | 0.84 |  |
| rs17677724 | 5 | 128015370 | T | C | 0.160 | 1.08(1.05-1.10) | 1.6×10^-9^ | +--+++++ | 0.16 |  |
| rs4073717 | 5 | 170864021 | T | G | 0.200 | 0.938(0.918-0.959) | 1.1×10^-8^ | -++++++- | 0.41 |  |
| rs35233 | 5 | 52290880 | A | G | 0.12 | 0.927(0.903-0.952) | 2.8×10^-8^ | ----+--+ | 0.55 | novel |
| rs1384547 | 6 | 44599680 | T | C | 0.335 | 0.936(0.919-0.954) | 2.0×10^-12^ | --+---+- | 0.21 |  |
| rs9475400 | 6 | 55638258 | T | C | 0.0987 | 1.12(1.08-1.15) | 1.2×10^-13^ | ++-+++++ | 0.78 |  |
| rs12209223 | 6 | 76164589 | A | C | 0.113 | 1.15(1.12-1.18) | 2.1×10^-23^ | +++++++- | 0.80 |  |
| rs189933136 | 8 | 130749375 | T | C | 0.818 | 1.12(1.09-1.15) | 2.7×10^-15^ | ++++++-+ | 0.56 |  |
| rs12350134 | 9 | 110430765 | T | G | 0.132 | 0.904(0.881-0.928) | 2.2×10^-14^ | -----++- | 0.79 |  |
| rs10817926 | 9 | 119483436 | A | C | 0.461 | 0.930(0.914-0.947) | 4.9×10^-16^ | -------+ | 0.75 |  |
| rs10983775 | 9 | 120521100 | T | C | 0.547 | 0.950(0.933-0.967) | 8.8×10^-9^ | -+-+--+- | 0.53 |  |
| rs12377624 | 9 | 129373110 | C | G | 0.357 | 0.938(0.921-0.955) | 9.5×10^-12^ | -------- | 0.94 |  |

GO, Genetics of Osteoarthritis; JPN, Japan; DDH, demelopmental dysplasia of the hip, CHR, chromosome; POS, position (position base pair in genome build hg19); Allele1, effect allele; Allele2, alternative allele; Freq1, frequency for effect allele across this analysis; OR, odds ratio; CI, confidence interval; P-value, meta-analysis p-value; Direction, summary of effect direction for each study(GO hip OA GWAS/Japanese hip dysplasia GWAS set1/Japanese hip dysplasia GWAS set2/Japanese hip dysplasia GWAS set3/Japanese dislocated hip GWAS set1/Japanese dislocated hip GWAS set2/Japanese dislocated hip GWAS set3/Japanese non-DDH hip OA GWAS); HetPVal, p-value for heterogeneity statistic

Supplementary table 16: Trans ethnic genetic correlation between JPN DDH and GO Hip OA.

| Genetic correlation between GO_HipOA and JPN_DDH calculated by popcorn* | | | | |
| --- | --- | --- | --- | --- |
| Val | (obs) | SE | Z | P |
| GO h1^2 | 0.026 | 0.002 | 11.040 | 0.000 |
| JPN h2^2 | 0.035 | 0.015 | 2.276 | 0.023 |
| pge | 0.536 | 0.189 | 2.455 | 0.014 |

GO, Genetics of Osteoarthritis; JPN, Japan; obs, the common-SNP observed scale heritability for both populations (GO and JPN); pge, the genetic effect correlation; SE, standerd error; Z, Z-score; P, p-value

Supplementary table 17: Partitioning heritability enrichment analysis with condition-specific chromatin accessibility data in chondrocytes and JPN DDH GWAS sumstats.

| Enrichment | Enrichment_se | Enrichment_p |
| --- | --- | --- |
| -0.00062 (0.16) | -0.13 (31.7) | 0.97 |

se, standard error

Supplementary table 18: lncRNAs located in the proximity of DDH GWAS association and regulated by allelic imbalance.

| lncRNA with AI | rsID | GWAS |
| --- | --- | --- |
| HCG25 | rs1704995 | Hip dysplasia |
| AL645940.1 |  |  |
| AC097518.2 | rs142273463 | DDH |
| FOXCUT | rs146711505 |  |
| AL512329.2 |  |  |
| AC008035.1 | rs78572420 |  |
| AC008014.1 |  |  |
| AC021752.1 | rs7168702 |  |
| MSTRG.19301.11 | rs143384 | Meta analysis JPN and UK DDH |
| CEP250-AS1 |  |  |

AI, Allelic imbalance; JPN, Japan; DDH, demelopmental dysplasia of the hip

Supplementary table 19: Minor allele frequencies of lead variants associated with DDH in the JPN GWAS across EAS and EUR (Non-Finnish) populations.

| SNP | EAS_MAF | EUR_MAF(non-Finish) |
| --- | --- | --- |
| rs142273463 | 0.0346 | 0.0 |
| rs146711505 | 0.276 | 0.343 |
| rs147057560 | 0.0215 | 1.11e-4 |
| rs78572420 | 0.229 | 0.0156 |
| rs7168702 | 0.0299 | 1.47e-5 |

EAS, East Asia; EUR, Europe

Supplementary table 20: Comparison of LD r^2^ between rs146711505 and EUR proxy variants in JPN and EUR populations.

| Japanese lead variants | UK proxy variants | EUR r^2^ | JPN r^2^ |
| --- | --- | --- | --- |
| s146711505 | rs2317961 | 0.918 | 0.291 |
|  | rs2816294 | 0.902 | 0.291 |
|  | rs7739648 | 0.90 | 0.283 |

EUR, Europe; JPN, Japan

Ⅳ. References

1. Purcell S, Neale B, Todd-Brown K, et al. PLINK: a tool set for whole-genome association and population-based linkage analyses. Am J Hum Genet 2007;81:559-75. doi:10.1086/519795
2. Terao C, Flanagan J, Tomizuka K, et al. Population-specific reference panel improves imputation quality and enhances locus discovery and fine-mapping. Research Square [Preprint]. August 07, 2023 https://doi.org/10.21203/rs.3.rs-3194976/v1
3. Koyama S, Liu X, Koike Y, et al. Population-specific putative causal variants shape quantitative traits. Nat Genet 2024 doi:10.1038/s41588-024-01913-5
4. Loh PR, Danecek P, Palamara PF, et al. Reference-based phasing using the Haplotype Reference Consortium panel. Nat Genet 2016;48:1443-48. doi:10.1038/ng.3679
5. Das S, Forer L, Schönherr S, et al. Next-generation genotype imputation service and methods. Nat Genet 2016;48:1284-87. doi:10.1038/ng.3656
6. 1000 Genomes Project Consortium; Auton A, Brooks LD, Durbin RM, et al. A global reference for human genetic variation. Nature. 2015 1;526(7571):68-74. doi: 10.1038/nature15393.
